# Assessing the authenticity and purity of a commercial *Bacillus thuringiensis* bioinsecticide through whole genome sequencing and metagenomics approaches

**DOI:** 10.3389/fmicb.2025.1532788

**Published:** 2025-01-31

**Authors:** Yari Van Laere, Marie-Alice Fraiture, Andrea Gobbo, Sigrid C. J. De Keersmaecker, Kathleen Marchal, Nancy H. C. Roosens, Kevin Vanneste

**Affiliations:** ^1^Transversal Activities in Applied Genomics, Sciensano, Elsene, Belgium; ^2^Department of Plant Biotechnology and Bioinformatics, UGent, Zwijnaarde, Belgium; ^3^Department of Information Technology, UGent, Zwijnaarde, Belgium

**Keywords:** *Bacillus thuringiensis*, bioinsecticide, whole genome sequencing, metagenomics, purity, authenticity

## Abstract

Biopesticides, biological agents for pest control in plants, are becoming increasingly prevalent in agricultural practices. However, no established methodology currently exists to assess their quality, and there are currently no publicly available authenticity and purity evaluations of commercial products. This lack of data may represent risks because of their widespread dispersal in the environment. We evaluated the potential of whole genome sequencing (WGS) and metagenomics approaches, including nanopore long-read sequencing, to verify both authenticity (i.e., the labeled strain) and biological purity (i.e., the absence of any undesired genetic material) of commercial *Bacillus thuringiensis* bioinsecticides. Four commercially available bioinsecticidal products containing *Bacillus thuringiensis* serovar *kurstaki* strain HD-1 were collected from the European market as a case study. Two sequencing approaches were employed: WGS of isolates and metagenomics sequencing of all genetic material in a product. To assess authenticity, isolate WGS data were compared against the publicly available reference genome of the expected strain. Antimicrobial resistance gene content, insecticidal gene content, and single nucleotide polymorphism differences were characterized to evaluate similarity to the reference genome. To assess purity, metagenomic sequencing data were analyzed using read classification and strain differentiation methods. Additionally, long- and short-read data were used to assess potential large-scale structural variations. Our results confirmed all investigated products to be authentic and pure. With the increasing usage of biopesticides, it is crucial to have adequate quality control methods. Our proposed approach could be adapted for other biopesticides, and similar products, providing a standardized and robust approach to contribute to biopesticide safety.

## 1 Introduction

Pesticide use has contributed considerably to increasing agricultural yields (Tudi et al., 2021). There exist however many drawbacks related to the use of conventional pesticides, such as pollution, bioaccumulation in people and wildlife, biodiversity losses, increase of secondary pests, and elimination of beneficial insects (Fenibo et al., 2021). Initiatives such as the Farm to Fork strategy, part of the European Green Deal, consequently aim to reduce the use of chemical pesticides (European Commission, 2020). This increases the demand for alternatives such as biopesticides, i.e., products comprised of active substances derived from, or containing, living organisms and certain minerals (Bourguignon, 2017). The use of biopesticides at a global scale increases by almost 10% every year, and the growth of the biopesticide market is projected to eventually outpace that of chemical pesticides (Damalas and Koutroubas, 2018).

One of these commonly used biopesticides is *Bacillus thuringiensis*, a Gram-positive, spore-forming bacterium, part of the *Bacillus cereus sensu lato* group, which produces several proteins that possess insecticidal activity. At the onset of sporulation, crystal (Cry) and/or cytolytic (Cyt) proteins are produced. When insects ingest these proteins, their digestive tract denatures the proteins and renders them soluble after which proteolytic activation of the toxic protein occurs. The toxin then binds the midgut epithelial cells and causes the formation of pores, resulting in cell lysis and death of the insect. During the vegetative growth phase of *B. thuringiensis*, some strains produce additional insecticidal proteins (Vip and Sip) (Palma et al., 2014). About 90% of the microbial biopesticides are derived from this bacterium (Kumar and Singh, 2015). One particular strain often used in commercialized products is *B. thuringiensis* serovar *kurstaki* strain HD-1 (Dulmage, 1970), also known as strain ABTS-351 (Alvarez et al., 2021). This strain is the active substance of multiple commercially available bioinsecticides such as Dipel DF, BioBit DF, Foray 48B, and Foray 76B, which are used in agriculture and forestry to protect against damage from Lepidoptera.

Since increasing bioinsecticide use results in large quantities of these products being released into the environment, they undergo regulation at the European level to ensure their safety (Bourguignon, 2017). For plant protection products to be sold on the European market, the active substances first need to be reviewed by the European Food Safety Authority (EFSA) as detailed in Regulation (EC) No 1107/2009. The conclusion of EFSA is then presented to the European Commission, which can approve it at the European level. An active substance approved at the European level can then be used in products that have to be authorized at the member state level. Regulation (EC) No 1107/2009 specifies that micro-organisms require, among others, taxonomic classification, a strain deposition in an internationally recognized culture collection, and information about their antimicrobial resistance genes, to acquire approval. *B. thuringiensis* serovar *kurstaki* strain HD-1 was last reviewed by EFSA for use as an active substance in plant protection products in 2021 (Alvarez et al., 2021). To this end, a confidential, annotated whole genome sequence was provided to EFSA. At the European level, the approval of this active substance was recently renewed (European Commission, 2023). For use as a biocidal product, an active substance has to be reviewed by the European Chemicals Agency (ECHA), as detailed in Regulation (EU) No 528/2012 to assess its safety and efficacy, before approval at the European level can be granted by the European Commission. *B. thuringiensis* serovar *kurstaki* strain HD-1 was reviewed by ECHA as an active substance for use in biocidal products in 2016 (ECHA, 2016), and approved at the European level in 2017 for 10 years (European Commission, 2016).

Once bioinsecticidal products with microbial active substances are on the market, there is no independent monitoring of their authenticity (i.e., the labeled strain of a product) or purity (i.e., the absence of any other undesired genetic material in the product). Furthermore, there are no publicly available authenticity and purity evaluations of commercial biopesticide products. The absence of post-market surveillance to ensure product quality can be explained partly by the lack of appropriate tools for analyzing microbial pesticides. Contaminated biopesticides could introduce pathogens into the food chain and/or into the environment, potentially posing a threat to public health or damaging ecosystems. They could contribute to the spread of antimicrobial resistance (AMR) genes in the environment through horizontal gene transfer (Tao et al., 2022). Furthermore, inauthentic biopesticides might not provide the desired results, leading to damaged crops and lower agricultural yields. Several cases have been documented in recent years of commercialized products not containing the labeled species or containing microbial contaminations, for instance in commercial fermentation products (D’aes et al., 2022) and probiotics (Dioso et al., 2020; Vermeulen et al., 2020). Current methods to analyze the authenticity and purity of fermentation and probiotic products often rely on analyzing small genomic regions using species-specific PCR and 16S rRNA or 23S rRNA gene analysis (Mazzantini et al., 2021). Members of the *B. cereus sensu lato* group are however genetically very similar, and PCR-based methods targeting single genes or variable regions of the 16S and 23S rRNA genes have failed to reveal consistent differences between its members (EFSA Panel on Biological Hazards (BIOHAZ), 2016). Strain-level classification for *B. thuringiensis* is even more challenging and according to the EFSA Panel on Biological Hazards (BIOHAZ) (2016), the only manner to unambiguously recognize a specific strain of *B. thuringiensis* is through the characterization of its genetic material by whole genome sequencing (WGS). WGS is often done by means of Illumina short-read sequencing, providing highly accurate but short (75–300 bp) reads that provide the necessary information to characterize a strain (Hasman et al., 2014). This technology has been used for the detection of (unauthorized) genetic modifications in commercial food enzyme products (D’aes et al., 2021), finding insecticidal genes in *B. thuringiensis* (Liu et al., 2021; Chung et al., 2024), and detecting AMR genes to verify the authenticity of the product. To accurately assess the purity, information is needed on the full genetic content of a product. This can be achieved through metagenomic sequencing, which involves extraction of genetic material directly from the product, followed by sequencing. Since there is no isolation, genetic material from all present organisms is sequenced, allowing for an assumption-free and unbiased analysis capable of capturing unculturable microorganisms. This approach is more expensive than targeted methods like PCR, ELISA, microarrays, or 16S sequencing. However, PCR requires the design and validation of primers and has a very low throughput. ELISA has the same limitations. Micro-arrays have a high throughput, but the development of the probes is very time-consuming. 16S sequencing has a very high throughput, but it still provides far less information. Shotgun metagenomics provides information down to the single nucleotide level, allowing the detection of relevant genes like AMR genes and virulence factor genes, which are important in the context of purity. Recent developments in metagenomics even allow for achieving strain-level resolution in foodborne outbreak investigation (Buytaers et al., 2020), aided by the advent of long-read sequencing technologies such as those offered by Oxford Nanopore Technologies. This technology offers longer sequencing reads (up to several 10 kb) that despite exhibiting a higher error rate compared to short-read sequencing, facilitate resolving complex genomic regions by, for instance, spanning long repetitive regions (Mahmoud et al., 2019). Combining short- and long-read sequencing technologies for metagenomics also enables the detection of impurities in microbial fermentation products (D’aes et al., 2022). Next generation sequencing has been used before to analyze the authenticity and purity of food products (Haynes et al., 2019), but its added value to study the authenticity and purity of microbial biopesticides has never been assessed before.

In this study, we present a methodology using genomics approaches to analyze the authenticity and purity of commercial *B. thuringiensis*-based microbial bioinsecticides, applied to four products collected on the European market where they are sold as a bioinsecticide containing *B. thuringiensis* serovar *kurstaki* strain HD-1 in the form of a powder. All products were analyzed using both isolate short-read sequencing, and metagenomics short and long-read sequencing, to provide a strain-level characterization and to enable the evaluation of their authenticity and purity. Our study provides a methodological framework to standardize quality control for biopesticides using genomics approaches. This approach could be adapted for other biopesticides and similar products.

## 2 Materials and methods

### 2.1 Sample collection

**Figure 1:**
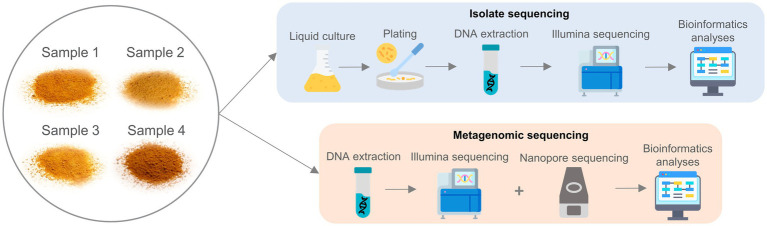
Workflow overview for the four *Bacillus thuringiensis* samples. Each of the four samples underwent both isolate and metagenomic sequencing. For isolate sequencing, each sample was cultured in liquid medium and subsequently plated. For every sample, four colonies were selected for DNA extraction and short-read sequencing, followed by bioinformatic analysis. For metagenomics sequencing, direct DNA extraction was performed for both short- and long-read sequencing on all four samples, followed by bioinformatics analysis.

Four samples were taken from four different commercial bioinsecticide products (i.e., one sample per product), collected by the French competent authorities (Service Commun des Laboratoires of The Ministry of Economics, Finance and Industrial and Digital Sovereignty of France) on the European market where they are sold as a bioinsecticide containing *B. thuringiensis* serovar *kurstaki* strain HD-1. The samples were collected in a powder form and were subjected to both isolate and metagenomics sequencing using short- and/or long-read sequencing. An overview of the sample processing workflow is available in Figure 1.

### 2.2 Isolate sequencing

For each individual sample, 500 mg of powder was added to 2 mL of Brain-Heart Infusion broth (Sigma-Aldrich) for overnight incubation at 30°C. From this microbial culture, 100 μL was plated on nutrient agar (Sigma-Aldrich) without antibiotics for overnight incubation at 30°C. For each individual sample, 4 isolates were selected for DNA extraction using the Quick-DNA™ HMW MagBead Kit (ZymoResearch). To this end, each microbial isolate was inoculated into 2 mL of Brain-Heart Infusion broth (Sigma-Aldrich) for an overnight incubation at 30°C. After centrifugation for 1 min at 5,000 g, the supernatant was discarded and the pellet was mixed with 100 μL of PBS (Gibco). After centrifugation for 1 min at 5,000 g, the supernatant was transferred to a new tube (referred to as Mix A) and the pellet was mixed with 1 mL of PBS (Gibco). After centrifugation for 1 min at 5,000 g, the supernatant was discarded. The pellet was dissolved in 100 μL of Tris–HCl 1 M (Invitrogen) and 20 μL of MetaPolyzyme (5 mg/mL; Sigma). After incubation at 37°C for 1 h, Mix A, 20 μL of 10% SDS (Thermo Fisher Scientific), and 10 μL of Proteinase K (20 mg/mL) were added. After an incubation at 55°C for 30 min, a centrifugation for 1 min at 5,000 g was applied. The supernatant was gently mixed at room temperature for 20 min with 800 μL of the Quick-DNA™ MagBinding Buffer and 33 μL of the MagBinding Beads. After magnetic bead separation, the supernatant was discarded and the pellet was gently resuspended in 500 μL of the Quick-DNA^™^ MagBinding Buffer and incubated at room temperature for 5 min. After magnetic bead separation, the supernatant was discarded and the pellet was gently mixed with 500 μL of the DNA Pre-Wash Buffer. After magnetic bead separation, the supernatant was discarded and the pellet was washed 2 times with 900 μL of the g-DNA Wash Buffer. After magnetic bead separation, the supernatant was discarded and the pellet was dried at 55°C for 7 min. The pellet was then mixed with 50 μL of the DNA Elution Buffer and incubated at 55°C for 10 min. After magnetic bead separation, the eluted DNA extract was transferred to a new tube. DNA concentration was measured using the Qubit 4 Fluorometer (Thermo Fisher Scientific). DNA purity was evaluated using the Nanodrop® 2000 (Thermo Fisher Scientific) through the A260/A280 and A260/A230 ratios. The DNA library was prepared using the Nextera XT DNA library preparation kit (Illumina) according to the manufacturer’s instructions. The sequencing was carried out on an Illumina MiSeq system with the V3 chemistry, obtaining 250 bp paired-end reads.

### 2.3 Metagenomic sequencing

DNA was extracted directly from the 4 samples using the Quick-DNA^™^ HMW MagBead Kit (ZymoResearch). For each individual sample, 100 mg was mixed with 100 μL of PBS (Gibco) and centrifuged for 1 min at 5,000 g. The supernatant was transferred to a new tube (Mix A). The pellet was dissolved in 1 mL of PBS (Gibco). After centrifugation for 1 min at 5,000 g, the supernatant was discarded. The pellet was dissolved in 100 μL of Tris–HCl 1 M (Invitrogen) and 20 μL of MetaPolyzyme (5 mg/mL; Sigma). After incubation at 37°C for 1 h, Mix A, 20 μL of 10% SDS (Thermo Fisher Scientific), and 10 μL of Proteinase K (20 mg/mL) were added. After an incubation at 55°C for 30 min, a centrifugation for 1 min at 5,000 g was applied. The supernatant was gently mixed at room temperature for 20 min with 800 μL of the Quick-DNA^™^ MagBinding Buffer and 33 μL of the MagBinding Beads. After magnetic bead separation, the supernatant was discarded and the pellet was gently mixed at room temperature for 5 min in 500 μL of the Quick-DNA^™^ MagBinding Buffer. After a magnetic bead separation, the supernatant was discarded and the pellet was gently mixed with 500 μL of the DNA Pre-Wash Buffer. After magnetic bead separation, the supernatant was discarded and the pellet was washed 2 times with 900 μL of the g-DNA Wash Buffer. After magnetic bead separation, the supernatant was discarded and the pellet was dried at 55°C for 7 min. The pellet was then mixed to 50 μL of the DNA Elution Buffer and incubated at 55°C for 10 min. After magnetic bead separation, the eluted DNA extract was obtained. The DNA extract was visualized using Tapestation 4200 and associated genomic DNA Screen Tape and reagents (Agilent) (see Supplementary Figure S1) to check for DNA integrity. The DNA concentration was measured using a Qubit 4 Fluorometer (Thermo Fisher Scientific). DNA purity was evaluated using the Nanodrop® 2000 (Thermo Fisher Scientific) through the A260/A280 and A260/A230 ratios.

For short-read sequencing, the DNA library was prepared using the Nextera XT DNA library preparation kit (Illumina) according to the manufacturer’s instructions. The sequencing was carried out on an Illumina MiSeq system with the V3 chemistry, obtaining 250 bp paired-end reads. For long-read sequencing, the DNA libraries were prepared using the ligation sequencing kit (SQK-LSK109; Oxford Nanopore Technologies) according to the manufacturer’s instructions. Each DNA library was loaded on an individual R9.4.1 MinION flow cell to be sequenced for 72 h.

### 2.4 Conventional PCR for chimeric cry gene

To check the potential existence of a chimeric *cry* gene observed in one of the isolates (see Results), a set of primers (F: GCTCAGGGCATAGAAGGAA; R: GAATCGGGGTTACAGAAGCA) was designed with the help of Primer3 (Untergasser et al., 2012) software to cover 766 bp of the chimeric *cry* gene. A conventional PCR assay was carried out using a standard 25 μL reaction volume including 1X Green DreamTaq PCR Master Mix (Thermo Fisher Scientific), 400 nM of each primer (Eurogentec), and 10 ng of DNA. The PCR program was applied on a Swift MaxPro Thermal Cycler (Esco) and was composed of 1 amplification cycle at 95°C for 1 min, 35 amplification cycles at 95°C for 30 s, at 60°C for 30 s, and at 72°C for 1 min and 1 final amplification cycle at 72°C for 5 min. The final PCR products were visualized by electrophoresis using the Tapestation 4200 device with the associated D1000 Screen Tape and reagents (Agilent) (see Supplementary Figure S2). The final PCR products were purified through USB ExoSAP-IT PCR Product Cleanup (Affymetrix) and sequenced on a Genetic Sequencer 3,500 using the Big Dye Terminator Kit v3.1 (Applied Biosystems). Alignment of the resulting sequences was done with Clustal Omega (Madeira et al., 2022) using the web interface of EBI with default parameters and the alignment was subsequently visualized in Jalview 2.11.2.7 (Waterhouse et al., 2009). The PCR assay was applied on DNA extracted either from sample 3 and sample 4, or from isolates 1–4 of sample 3 and isolates 1–4 of sample 4. In each PCR assay, a no template control was included.

### 2.5 Data analysis

**Figure 2:**
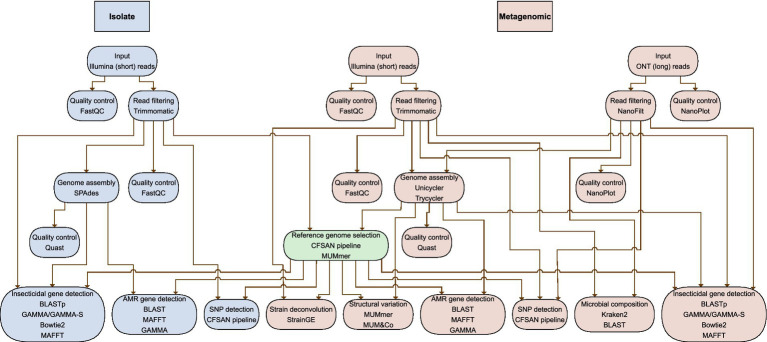
Schematic representation of the data analysis workflow. Each node describes an analysis step and lists the main tools used for that step. The blue section shows the analyses on the isolate sequencing data, while the orange section shows the analyses on the metagenomic sequencing data. The green node uses both isolate and metagenomic sequencing data. Both the orange and blue sections start with quality control and filtering of low-quality reads. After this, (meta)genome assemblies are made, which are also subjected to quality control. The pre-processed isolate sequencing data and one of the hybrid metagenomic assemblies are used to select a reference genome. (meta)genome assemblies, pre-processed sequencing data, and the reference genome are used to perform in-depth characterization of the samples, as indicated in the lowest row.

An overview of the entire bioinformatics workflow for both short-read isolate data, and short- and long-read metagenomics data is presented in Figure 2 and described in each of the sections below.

#### 2.5.1 Isolate genome assembly

Short reads were trimmed and filtered using Trimmomatic 0.38 (Bolger et al., 2014) with the following settings: “ILLUMINACLIP” set to NexteraPE-PE.fa:2:30:10, “LEADING” set to 10, “TRAILING” set to 10, “SLIDINGWINDOW” set to 4:20 and “MINLEN” set to 50. Only paired reads were retained for analyses. The quality of the raw and pre-processed reads was evaluated with FastQC 0.11.7 (Babraham Bioinformatics, 2018) with default settings. Processed reads were assembled using SPAdes 3.15.3 (Bankevich et al., 2012) with default settings. Assembly statistics were obtained with Quast 5.0.2 (Gurevich et al., 2013).

#### 2.5.2 Metagenomic genome assembly

Short reads were trimmed and filtered with Trimmomatic and the quality of raw and pre-processed reads was assessed with FastQC as described in Section 2.5.1. Long reads were basecalled with Guppy 6.4.6 (Oxford Nanopore Technologies, 2024). All basecalling was done in GPU mode with the super accurate (sup) model. Long reads were filtered with NanoFilt 2.8.0 (De Coster et al., 2018) to remove reads shorter than 1,000 bp and with a mean Phred score lower than 7. The quality of the raw and pre-processed reads was assessed with NanoPlot 1.36.2 (De Coster et al., 2018) with default settings. Hybrid metagenome assembly using a “long reads first” approach was first attempted with Trycycler 0.5.4 (Wick et al., 2021) for all samples, but only succeeded for sample 2. The methodology for this assembly is detailed in Supplementary Text S1. To create a successful Trycycler assembly, strict filtering is required. As not enough reads remained after strict filtering for samples 1, 3, and 4, hybrid assemblies were created with Unicycler 0.5.0 using a “short reads first” approach (Wick et al., 2017), using default settings and SPAdes 3.15.3, Racon 1.3.1 (Vaser et al., 2017), and BLAST+ 2.13.0 (Camacho et al., 2009) as dependencies. Long-read polishing was performed with Medaka 1.7.3 (nanoporetech, 2023) using the r941_min_sup_g507 model. A first round of short-read polishing was done with Polypolish 0.5.0 (Wick and Holt, 2022) using default settings, using the pre-processed metagenomic short reads. Read mapping for Polypolish was done with bwa 0.7.17 (Li, 2013). A second round of short-read polishing was done using the same pre-processed metagenomic short reads with the polka.sh script of the MaSuRCA 4.1.0 (Zimin and Salzberg, 2020) toolkit using default settings. Assembly statistics were obtained with Quast 5.0.2 for all hybrid assemblies.

#### 2.5.3 Reference genome selection

Since the genome sequence used by EFSA is confidential, we looked for a suitable reference genome by downloading *B. thuringiensis* assemblies from GenBank. To include assemblies that were not explicitly labeled as HD-1, HD1, or ABTS-351, the search also included strains labeled as Dipel, BioBit, or Foray, since these are known to contain *B. thuringiensis* serovar *kurstaki* strain HD-1. This resulted in 5 assemblies. Two assemblies were classified as *B. thuringiensis* serovar *kurstaki* strain HD-1 (accessions GCA_000717535.1 and GCA_000710255.1), one as *B. thuringiensis* strain ABTS-351 (accession GCA_020809105.1), one as *B. thuringiensis* strain dipel (accession GCA_025210105.1) and one as *B. thuringiensis serovar kurstaki* strain HD 1i (accession GCA_000835235.1). In particular, assembly GCA_000710255.1 was created to provide more insights into *B. thuringiensis* serovar *kurstaki* strain HD-1 used as a biocontrol agent and originated from a reference isolate designated as the primary US reference standard (Day et al., 2014). Only three of the five assemblies, GCA_000717535.1, GCA_020809105.1, and GCA_000835235.1 were listed as having an assembly level “complete genome.” The dnadiff function of MUMmer 4.0.0 (Marçais et al., 2018) was used to estimate the number of single nucleotide polymorphisms (SNPs) between the five assemblies and the high-quality Trycycler assembly created previously for sample 2. Additionally, the total SNP distance between all short-read sequencing data (for both isolates and metagenomic samples) was estimated by using the CFSAN SNP pipeline 2.0.2 with default settings, retaining the filtered SNPs (Davis et al., 2015). Each of the five assemblies was used as a reference genome for the CFSAN SNP pipeline separately. Two reference genomes were selected as being the best reference genomes, including the primary US reference for *B. thuringiensis* serovar *kurstaki* strain HD-1 (see Results), although we cannot exclude with full certainty the possibility that the reference strain in the dossier contained some genetic differences to this primary US reference (see Discussion).

#### 2.5.4 Isolate characterization

Genotypic AMR detection was performed using the BLAST-based method described previously (Bogaerts et al., 2021), with one modification, i.e., the National Database of Antibiotic Resistant Organisms (NDARO) (retrieved on 2023-09-24) was used instead of the ResFinder database. Hits with >80% sequence identity and > 80% sequence coverage were retained. Multiple sequence alignment was done with MAFFT 7.475 (Katoh and Standley, 2013) with the --adjustdirection option to compare the found AMR gene sequences at the nucleotide level against each other. Completeness of open reading frames (ORFs) was assessed with GAMMA 2.2 (Stanton et al., 2022) using a 50% identity threshold, comparing the found AMR genes to the sequences from the NDARO database.

Insecticidal gene detection was done by first translating the selected reference (see Section 2.5.3) to amino acid sequences with the SeqKit 2.10 (Shen et al., 2016) translate function, using translation table 11 for all six frames. The insecticidal gene content of the reference genome was determined by performing a BLASTp (BLAST+ 2.13.0) search with an identity threshold of 95% against the Bacterial Pesticide Protein Resource Center (BPPRC) database (Panneerselvam et al., 2022). For each genomic location with hits, there were multiple hits with varying lengths. Only the longest hits were kept, which still resulted in multiple hits with identical length for some genomic locations. For these hits, the nucleotide sequences were extracted and compared to the reference genome. Only hits with an identical nucleotide sequence were kept. For some genomic locations, this still resulted in multiple hits in which case a hit associated with *B. thuringiensis* serovar *kurstaki* strain HD-1 in literature was chosen if this existed. An overview of the literature on the insecticidal genes in *B. thuringiensis* serovar *kurstaki* strain HD-1 can be found in Supplementary Text S2. If none of the remaining hits had a link to *B. thuringiensis* serovar *kurstaki* strain HD-1 in literature, the hit with the lowest fourth rank was arbitrarily chosen [e.g., *cry2Aa1* and *cry2Aa9* are two names for the exact same protein sequence, in which case *cry2Aa1* was selected because it has a lower fourth rank (one vs. nine)]. Once the insecticidal genes in the reference genome were found, the isolate assemblies were translated to amino acid sequences with SeqKit 2.10 as mentioned before. Next, GAMMA-S 2.2 was used to look for the protein sequences corresponding to the insecticidal genes from the reference genome in the isolate assemblies using a minimum sequence identity of 85%. A multiple sequence alignment with the nucleotide sequences that correspond with the detected protein sequences in the isolates and the reference genome was done with MAFFT 7.475, using the --adjustdirection option. This step however only provided information on the insecticidal protein sequences found in the reference genome. To look for additional insecticidal protein sequences in the isolate assemblies, GAMMA-S was also used with the BPPRC database to check if any insecticidal protein sequences could be found in other locations. To complement the information from the assembly-based detection, the pre-processed reads were mapped against the reference genome with Bowtie2 2.4.1 (Langmead and Salzberg, 2012) using the --end-to-end and --sensitive options. The mapping results were visually inspected using the Integrative Genomics Viewer 2.12.3 (Robinson et al., 2011).

Lastly, the CFSAN SNP pipeline 2.0.2 was used to create a SNP matrix using the pre-processed reads of all 16 isolates and the reference genome selected as described in Section 2.5.3.

#### 2.5.5 Metagenomic characterization

Taxonomic classification was performed for all short- and long-read metagenomic sequencing data of all four samples using Kraken 2 2.1.1 (Wood et al., 2019) using an in-house constructed database containing all NCBI RefSeq “Complete genome” entries (database accessed on the 11th of February 2021) with accession prefixes NC, NW, AC, NG, NT, NS, and NZ of the following taxonomic groups: archaea, bacteria, fungi, human, protozoa, and viruses. This database also contained a selection of metazoan model species reference genomes, which are listed in Supplementary Table S1. Results were visualized using Krona 2.7 (Ondov et al., 2011). Any genus other than *Bacillus* present at a relative abundance of ≥1% reads was considered a contaminant. Reads classified as “other root” (i.e., reads that contain k-mers from different domains of life) were assembled using SPAdes 3.15.3 using the --meta option for the short reads and Flye 2.9.1 for the long reads using the --meta and --nano-hq options. Assembled contigs resulting from the “other root” read fraction were then manually analyzed using BLAST+ 2.7.1 against the NCBI nucleotide database.

To perform strain level characterization, StrainGE 1.3.7 (van Dijk et al., 2022) was used on the short read data. A database was created with all *Bacillus* genomes with assembly level “complete genome” from RefSeq (database accessed on the 18th of May 2022) using default settings. StrainGE consists of a two-step process, whereby StrainGST first reports one or more reference genomes from a database that are most similar to the strain(s) in a sample. StrainGR then identifies variants in the sample compared to the reference genomes found by StrainGST. StrainGST was run for 10 iterations. For follow-up analysis with StrainGR, the previously selected reference genome instead of the StrainGST output was used because it is the most suitable assembly for this strain and our sequencing data. Read alignment of the short reads to the reference genome was done using bwa 0.7.17 (Li, 2013), setting the -I parameter to 500. Sorting and indexing the resulting bam file was done using Samtools 1.9 (Danecek et al., 2021). StrainGR was run using default parameters. The nucmer and mummerplot functions of MUMmer 4.0.0 were used to visualize large structural variations. The metagenomic assemblies were mapped against the bacterial chromosome of the reference genome and mummerplots were constructed keeping only the contigs that aligned to the bacterial chromosome of the reference. To look for smaller structural variations, MUM&Co 3.8 (O’Donnell and Fischer, 2020) was used to compare the hybrid metagenomic assemblies against the reference genome, setting a genome length of 6.8 Mb. To annotate the found structural variants, both the reference genome and the metagenomic assemblies were annotated using Bakta 1.9.1 (Schwengers et al., 2021) with “Bacillus” for the genus parameter, “thuringiensis” for the species parameter, “HD-1” for the strain parameter, “+” for the gram parameter, and with the “keep-contig-headers” and “compliant” options enabled. Prodigal 2.6.3 (Hyatt et al., 2010) was used to make a prodigal training file from the reference genome.

Since the taxonomic classification and strain-level characterization indicated the samples to be extremely pure and only contain the labeled strain without inter- or intra-species contaminations (see Results), genotypic AMR detection and insecticidal gene detection were done as described in section 2.5.4 with the following two changes. First, the hybrid metagenome assemblies were used instead of the isolate assemblies. Second, minimap2 2.24 (Li, 2018) was used with the map-ont preset to allow mapping long reads to look for insecticidal genes. Lastly, SNP detection was done as described in Section 2.5.4 but using the short read datasets generated on the metagenome samples instead of isolate short read datasets.

## 3 Results

### 3.1 Evaluation of sequencing quality and selection of reference genome

Sequencing metrics indicated that all datasets were of high quality and could be retained for further analysis. A more detailed overview of the sequencing quality can be found in Supplementary Text S3. A suitable reference genome for further characterization was obtained by comparing publicly available *B. thuringiensis* serovar *kurstaki* strain HD-1 genome assemblies against the Trycycler assembly for sample 2 and against the isolate and metagenomic short-read sequencing data. The total number of SNPs of the Trycycler assembly compared to the publicly available genomes obtained by using MUMmer dnadiff is provided in Supplementary Table S6. Additionally, the sum of all SNPs found using the CFSAN pipeline on short-read datasets for both isolates and metagenomes against the publicly available genomes is provided in Supplementary Table S7. These analyses indicated that genome GCA_000835235.1, with 44 and 10 SNPs identified by MUMmer dnadiff and the CFSAN SNP pipeline, respectively; and genome GCA_000710255.1 with 39 and 11 SNPs identified by MUMmer dnadiff and the CFSAN SNP pipeline, respectively, were the closest to the samples. Since genome GCA_000710255.1 was fragmented and not complete, genome GCA_000835235.1 was considered the best reference genome. The third closest reference genome was GCA_025210105.1 with 153 and 40 SNPs identified by MUMmer dnadiff and the CFSAN SNP pipeline, respectively.

### 3.2 Characterization of the *Bacillus thuringiensis* samples by means of isolate short-read whole genome sequencing

#### 3.2.1 Isolate identification

Four isolates were collected for each of the four samples. Isolates were identified using the CFSAN SNP pipeline by comparing all 16 isolate short-read datasets against each other and the selected reference genome for *B. thuringiensis* serovar *kurstaki* strain HD-1. The resulting SNP matrix is presented in Table 1. The SNP distances to the reference genome were very small, with a maximum distance of four SNPs of any isolate to the reference genome. Moreover, the distance between individual isolates was also very limited with a maximum of five SNPs between any two isolates. These results confirmed that all isolates were *B. thuringiensis* serovar *kurstaki* strain HD-1.

**Table 1:** Pairwise SNP matrix comparing all isolate sequencing data to each other and the *B. thuringiensis* serovar *kurstaki* strain HD-1 reference genome.

	**Reference**	**S1-isl1**	**S1-isl2**	**S1-isl3**	**S1-isl4**	**S2-isl1**	**S2-isl2**	**S2-isl3**	**S2-isl4**	**S3-isl1**	**S3-isl2**	**S3-isl3**	**S3-isl4**	**S4-isl1**	**S4-isl2**	**S4-isl3**	**S4-isl4**
**Reference**	0																
**S1-isl1**	2	0															
**S1-isl2**	1	1	0														
**S1-isl3**	4	3	2	0													
**S1-isl4**	2	1	0	2	0												
**S2-isl1**	1	1	0	2	0	0											
**S2-isl2**	1	1	0	2	0	0	0										
**S2-isl3**	3	4	3	5	3	3	3	0									
**S2-isl4**	1	1	0	2	0	0	0	3	0								
**S3-isl1**	0	1	0	2	0	0	0	3	0	0							
**S3-isl2**	1	1	0	2	0	0	0	3	0	0	0						
**S3-isl3**	4	3	2	0	2	2	2	5	2	2	2	0					
**S3-isl4**	2	1	0	2	0	0	0	3	0	0	0	2	0				
**S4-isl1**	2	1	0	2	0	0	0	3	0	0	0	2	0	0			
**S4-isl2**	3	3	2	0	2	2	2	5	2	2	2	0	2	2	0		
**S4-isl3**	2	1	0	2	0	0	0	3	0	0	0	2	0	0	2	0	
**S4-isl4**	2	2	1	3	1	1	1	4	1	1	1	3	1	1	3	1	0

Column and row headers contain the isolate names, e.g., S1-isl1 stands for sample 1, isolate1. The values indicate the pairwise SNP distances between the corresponding samples.

#### 3.2.2 AMR gene characterization

Genotypic AMR detection results are presented in Table 2 and indicated that six AMR genes were present in the reference genome. Additional analysis with GAMMA showed that all AMR genes contained one or more amino acid substitutions compared to the NDARO reference sequences, for which results are presented in Supplementary Table S8. All AMR genes contained at least one amino acid substitution; *bla*, *bla2*, and* fosB/fosBx1* only contained amino acid substitutions, whereas *satA*, *blaIII,* and *vanZ-F* also contained indels, which caused truncations in *satA* and *vanZ-F*. The same six genes were detected in all four isolates for all four samples and no additional AMR genes were found. Moreover, multiple sequence alignment with MAFFT showed that the nucleotide sequences for all six AMR genes were identical in all isolates compared to the reference genome.

**Table 2:** AMR genes detected in the isolate and metagenomic assemblies.

Locus	%Identity	Hit length/locus length	Antibiotic	Accession
*bla*	97.50	921/921	Beta-Lactam	NG_047482.1
*satA*	92.77	553/555	Streptothricin	NG_064661.1
*blaIII*	88.09	966/951	Beta-Lactam	NG_148591.1
*fosB/fosBx1*	99.28/99.28	417/417/417	Fosfomycin/Fosfomycin	NG_055636.1/NG_050591.1
*bla2*	93.29	775/774	Carbapenem	NG_056058.1
*vanZ-F*	82.4	591/621	Vancomycin	NG_048535.1

The first column shows the found AMR gene locus. The second column shows the percentage identity between the sequence in the NDARO database and the sequences found in the isolate and metagenomic assemblies. The third column shows the length of the found sequences and the length of the locus in the NDARO database. The fourth column lists the antibiotics against which the loci confer resistance according to the NDARO database. The fifth column gives the GenBank accession for the NDARO sequence. Note that *fosB* and *fosBx1* were both found at the same genomic location with exactly the same sequence identity so that no best hit could be selected. More specific information on the nature of found mutations compared to the NDARO database, are presented in Supplementary Table S8.

#### 3.2.3 Insecticidal gene characterization

**Figure 3:**
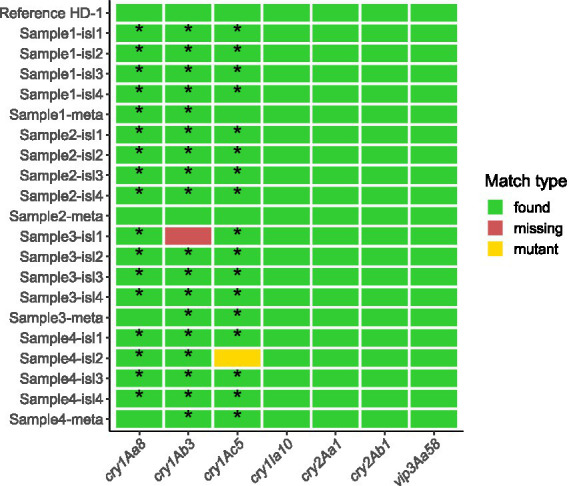
Insecticidal gene content of the isolate and metagenomic assemblies. Rows correspond to the reference genome, isolate (suffix “-isl”), or metagenomic (suffix “-meta”) assemblies, and columns correspond to the seven insecticidal genes present in the reference genome. For each assembly and insecticidal gene sequence, one of three match types can apply. A “found” match type indicates that the nucleotide sequence was entirely present in the isolate with 100% identity compared to the nucleotide sequence of the reference genome. A “missing” match type indicates that the sequence could not be found. A “mutant” match type indicates that an insecticidal gene sequence was found but with mutations. An asterisk presents that a gene with match type “found” was initially not found in the assemblies, but detected upon further investigation with read mapping.

Detection of insecticidal genes in the reference resulted in seven different genes that were found, always full-length and with 100% identity compared to the BPPRC database. Six of the insecticidal protein sequences (Vip3Aa58, Cry2Ab1, Cry2Aa1, Cry1Ia10, Cry1Ac5, and Cry1Aa8) were all encoded on the same plasmid in the reference genome (accession: NZ_CP009999.1). The protein sequence for Cry1Ab3 was encoded on a different plasmid (accession: NZ_CP010003.1). The results of the insecticidal gene detection in the 16 isolate assemblies are shown in Figure 3. All isolate assemblies contained a full-length copy of the protein sequences of Vip3Aa58, Cry2Ab1, Cry2Aa1, and Cry1Ia10 with 100% sequence identity compared to the amino acid sequences found in the reference genome. Multiple sequence alignment with the corresponding nucleotide sequences confirmed that these sequences were also identical at the nucleotide level. Most assemblies also contained partial but 100% identity hits for the Cry1Ac5, Cry1Ab3, and Cry1Aa8 protein sequences with the exception of two cases. No Cry1Ab3 sequence was detected for isolate 1 of sample 3, and the Cry1Ac5 protein sequence for isolate 2 of sample 4 contained amino acid substitutions. The assemblies were too fragmented to determine if Vip3Aa58, Cry2Ab1, Cry2Aa1, Cry1Ia10, Cry1Ac5, and Cry1Aa8 were encoded on the same plasmid. Read mapping of individual isolate short read datasets against the reference genome was performed to manually investigate the presence of *cry1Ac5*, *cry1Ab3*, and *cry1Aa8* (see Supplementary Figures S3–S14). This analysis indicated that partial hits for these genes were due to the genes being located on contig edges due to assembly fragmentation and confirmed that the full genes were present in the sequencing reads. Investigation of the missing *cry1Ab3* sequence for isolate 1 of sample 3 indicated that its host plasmid is likely present at a very low copy number (see Supplementary Figure S10). Investigation of the altered Cry1Ac5 protein sequence for isolate 2 of sample 4 confirmed the presence of several SNPs in the insecticidal gene. To ensure no additional insecticidal genes were present in any isolate on top of those seven present in the reference genome, an additional search was done in all isolate assemblies using all protein sequences in the BPPRC database, but no additional insecticidal genes were found.

The sequence of the altered *cry1Ac5* gene in isolate 2 of sample 4 corresponded with a different *cry* gene, *cry1Ab3*, which is present on a different plasmid, suggesting a chimeric sequence was present in this isolate. To confirm this chimeric change to be really present and not constitute an artifact, PCR amplification and Sanger sequencing of the *cry1Ac5* gene were performed for all isolates of samples 3 and 4, as well as directly on samples 3 and 4. These results confirmed the presence of a chimeric gene in isolate 2 of sample 4, comprising the beginning of *cry1Ac3* and the ending of *cry1Ac5* (see Supplementary Text S4). This chimeric construct was not present in any other isolates nor in the sequences obtained without isolation from samples 3 and 4. Additional investigation confirmed it was also not present in the reference genome (results not shown).

### 3.3 Characterization of the *Bacillus thuringiensis* samples by means of metagenomic short- and long-read sequencing

#### 3.3.1 Microbial composition

For all four samples, direct metagenomics sequencing of all DNA contained in a sample was also performed with both short- and long-read technologies. Taxonomic classification on both short- and long-read datasets was performed with Kraken2. An overview of the observed microbial composition of the samples is available in Supplementary Figures S15–S22, and a summary is presented in Supplementary Table S9. None of the samples contained genera with ≥1% reads assigned to it other than *Bacillus*. All samples contained an “other root” fraction of more than 1%, corresponding to reads containing k-mers belonging to different domains, which were assembled and manually compared to NCBI’s nucleotide database. This analysis indicated that contigs originating from the “other root” fractions only contained hits for the *B. thuringiensis* chromosome, *B. thuringiensis* plasmids, and *Bacillus* phages (results not shown). Evaluation of the microbial composition hence indicated no contaminating genera were present in the samples (excluding *Bacillus* phages).

#### 3.3.2 Strain-level deconvolution

Kraken2 demonstrated that the samples only contained bacteria belonging to the genus *Bacillus*. To identify which members of this genus were present and perform strain-level deconvolution, StrainGE was used using all publicly available complete *Bacillus* genomes from RefSeq as the underlying database and the metagenomic short-read data. In total, 904 complete *Bacillus* genomes from RefSeq were collected and clustered, resulting in 538 clusters. StrainGST demonstrated that all samples only contained one cluster containing the strain of interest, *B. thuringiensis* serovar *kurstaki* strain HD-1, and no other clusters were identified in any of the samples. According to StrainGR, the mean average callable nucleotide identity values for all replicons were > 99.99% for all samples, indicating very high similarity to the *B. thuringiensis* serovar *kurstaki* strain HD-1 reference cluster.

Given the observed purity of metagenomic samples, similar to isolates, the CFSAN pipeline was used to evaluate SNP distances between the metagenome samples using the short reads. The resulting SNP matrix indicated that the SNP distances were very small, with a maximum distance of any sample to the reference genome of one SNP, and no SNPs between any of the four metagenomic samples. Fewer SNPs were found here compared to the isolate sequencing data because SNPs are diluted more, causing them to become filtered during variant calling with the CFSAN pipeline.

#### 3.3.3 Evaluation of structural variation

**Figure 4:**
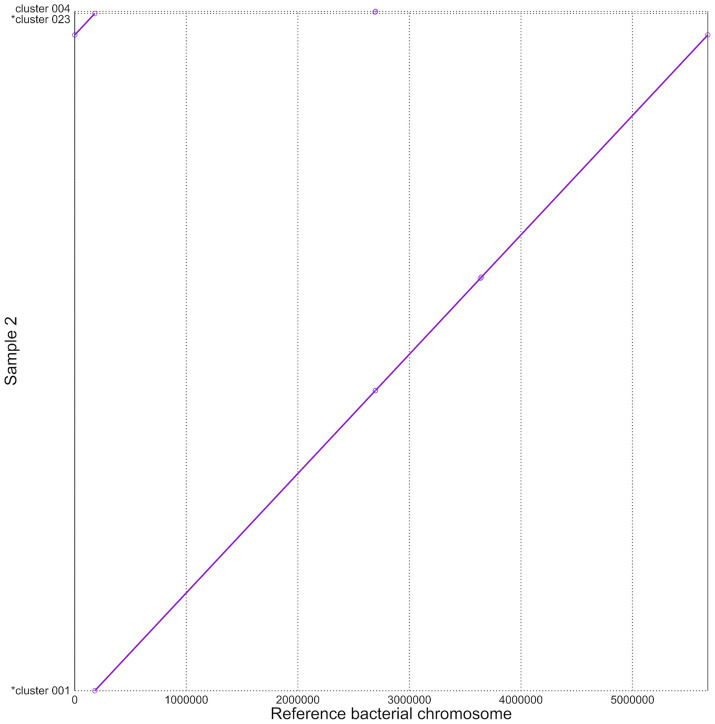
Mummerplot to assess large-scale structural variation in the Trycycler assembly of sample 2. The y-axis shows the Trycycler assembly of sample 2. The x-axis shows the bacterial chromosome of the reference genome *B. thuringiensis* serovar *kurstaki* strain HD-1 (NCBI accession: NZ_CP010005.1), with the labels corresponding to genomic locations in bp. The purple line indicates regions of homology between the reference and assembly in the same orientation. The smaller circles indicate the presence of small-scale structural variations. The Trycycler assembly corresponds almost completely with the bacterial chromosome of the reference, without any large-scale deletions, duplications or inversions detected. The small ‘nick’ at the origin is caused by the Trycycler assembly starting at position 180,076, with positions 1–180,076 attached to the end of the assembly. Note that plasmids were not considered in this visualization.

To look for structural variation, mummerplots were created to visually explore potential large-scale genomic rearrangements. A representative example is provided in Figure 4 for the Trycycler assembly of sample 2. Mummerplots for the other three samples are available in Supplementary Figures S23–S25. Genome assemblies were compared to the bacterial chromosome of the reference genome. Plasmids from the reference genome were not taken into account. For sample 2, a full bacterial chromosome is present that aligns with the bacterial chromosome of the reference genome. Although the assemblies of the other samples are more fragmented, a clear alignment to the bacterial chromosome of the reference genome is similarly present. Although some smaller structural variations were found in all assemblies, no evidence of any large-scale structural variations was detected. MUM&Co was used to look for smaller structural variations and did not identify any structural variations in the Trycycler assembly of sample 2, representing the highest-quality assembly. For the other samples, some smaller structural variations were identified. In sample 1, 13 mobile deletions and 2 contractions were found, mainly in hypothetical proteins and phages/transposases, but also in some predicted protein-coding genes. In sample 3, 3 mobile deletions and 3 contractions were found, mainly in protein-coding genes. In sample 4, 3 mobile deletions and 1 contraction were found, mainly in hypothetical proteins. Full results for all metagenomic samples can be found in Supplementary Tables S10–S13.

#### 3.3.4 AMR gene characterization

Genotypic AMR detection in the metagenomic samples using the hybrid assemblies resulted in the same six genes found in the reference genome, for which an overview is presented in Table 2. Similar to the isolate assemblies, the six genes in the metagenome assemblies were identical to each other and the reference genome. Consequently, the same amino acid substitutions as described in section 3.2.2 for the 16 isolates were also found in the four metagenome samples.

#### 3.3.5 Insecticidal gene characterization

Results of the insecticidal gene detection in the metagenomic assemblies are shown in Figure 3. Similar to the isolate assemblies, all metagenome hybrid assemblies contained a full-length copy of the protein sequences for Cry1Ia10, Cry2Aa1, Cry2Ab1, and Vip3Aa58 with 100% sequence identity compared to the amino acid sequences found in the reference genome. Multiple sequence alignment of the nucleotide sequences corresponding to these protein sequences confirmed that these genes were also identical at the nucleotide level. The assembly of sample 2, the highest-quality assembly created with Trycycler, also contained the protein sequences for Cry1Aa8, Cry1Ab3, and Cry1Ac5. The Unicycler assemblies for samples 1 and 4 however missed the Cry1Ab3 protein sequence and sample 3 had a partial hit with mutations for it. The assemblies for samples 3 and 4 contained partial but 100% identity hits for the Cry1Ac5 protein sequence, which were typically located on contig edges. The assembly for sample 1 contained a partial but 100% identity hit for the Cry1Aa8 protein sequence which was not located on a contig edge. In samples 1 and 2, Vip3Aa58, Cry2Ab1, Cry2Aa1, Cry1Ia10, Cry1Ac5, and Cry1Aa8 were encoded on the same plasmid with length 317,333 bp and 317,322 bp, respectively. The plasmid in the reference genome that contains the corresponding insecticidal genes is 317,336 bp long. In sample 2, Cry1Ab3 was encoded on a 69,354 bp plasmid. In the reference genome, the corresponding gene is found on a 69,317 bp plasmid. The other assemblies were too fragmented to find complete plasmids containing insecticidal genes. Read mapping of individual metagenomic short- and long-read datasets against the reference genome was performed to manually investigate the presence of *cry1Ab3*, *cry1Ac5*, and *cry1Aa8*. Full copies of all three genes could be found in all samples (see Supplementary Figures S26–S31). An additional read mapping was performed to evaluate the partial hit in sample 1 not located on a contig edge for *cry1Aa8*. The short and long reads were mapped against the hybrid assembly of sample 1, but no evidence was found for the presence of a partial copy of *cry1Aa8*, indicating that the partial hit was most likely due to a mis-assembly (see Supplementary Figure S32). The same approach was done for sample 1 to look for the presence of a partial copy of *cry1Ab3*, but no evidence of a partial copy of *cry1Ab3* was found (see Supplementary Figure S33). An additional search was done for all protein sequences in the BPPRC database in all metagenomic assemblies to look for additional locations with insecticidal protein sequences, but no additional insecticidal genes were found.

## 4 Discussion

In this study, we proposed for the first time a strategy to investigate the authenticity and purity of commercial microbial bioinsecticides, using four products labeled as containing *B. thuringiensis* serovar *kurstaki* strain HD-1 as a proof-of-concept. In particular, we present a methodological approach that uses WGS and metagenomics approaches, including long-read sequencing, to extensively investigate both the authenticity and purity of the commercial bioinsecticide samples. The technologies used in our study are not new and similar methodologies have been used in different, related fields. The novelty of our approach lies in the application of these technologies, like metagenomics, to analyze for the first time the authenticity and purity of microbial bioinsecticides. In particular, we demonstrate that these technologies, which do not have widespread adoption in the context of biopesticide quality control, can be of substantial added value for these kinds of samples.

To evaluate authenticity, we verified whether the commercial products contained the specific bacterial strain on the label and not another variant of *B. thuringiensis*, or even another species. Evaluating purity required verifying whether the products did not contain other contaminations, and whether no other *B. thuringiensis* strains were present in minor quantities. *B. thuringiensis* is part of the *B. cereus* group, with their *cry* or *cyt* gene-carrying plasmids acting as a species-defining feature (Biggel et al., 2022). To perform strain-level identification, strain-specific PCR could be used, but there exist several hundred insecticidal proteins, making this approach highly laborious and inefficient, as several custom PCR assays would need to be developed and validated (De Bock et al., 2021). Moreover, targeted assays can only capture information on their intended genetic target, and will therefore miss other genetic alterations in the genome of the targeted strain, as well as miss reporting on any other contaminating species. In this study, we alternatively used both isolate and metagenomic sequencing, incorporating the use of long-read sequencing, to explore whether (meta)genomics methods can provide information on authenticity and purity. WGS of isolates is a relatively cost-effective and fast method that allows thorough characterization of a single strain (Hasman et al., 2014). When using short-read sequencing, the resulting genome assemblies are often very fragmented, but still provide enough information to allow characterization about the sample, eliminating the need for prior knowledge (Chen et al., 2022). By utilizing long-read sequencing for metagenomic analysis, the disadvantages of using short reads for resolving repetitive regions can be overcome, enabling the detection of structural variation (Ho et al., 2020) and making the detection of genetic modification possible (Buytaers et al., 2021). This approach is however currently less established with fewer guidelines and recognized procedures, and is also more expensive.

To analyze authenticity, a good reference is required to allow the comparison of samples. To find a reliable reference, multiple publicly available genomes were assessed, for which two suitable candidates were found. One of these two was designated as the primary US reference standard for *B. thuringiensis* (Day et al., 2014). Since this genome was however fragmented, we alternatively employed a reference genome for which the chromosome was fully scaffolded to allow evaluating structural variation. Additional investigation demonstrated that this genome was extremely similar to the US reference standard (see Supplementary Text S2). Application of our approach for assessing authenticity of other products, will consequently also necessitate the existence of a suitable reference for comparison. Isolate sequencing confirmed, using SNP-based methods, that all four isolates for all four samples were *B. thuringiensis* serovar *kurstaki* strain HD-1 and no other species nor strains were encountered. Isolates from different products were found to differ very few SNPs, indicating that these biopesticide products are remarkably stable. All 16 isolates contained exactly the same AMR genes as the reference, and these genes were always identical down to the single nucleotide level. Since none of the found AMR genes corresponded exactly to the NCBI NDARO database, we also investigated whether the ORFs of these genes were intact. All detected AMR genes had at least one amino acid substitution and two had truncations. According to the EFSA and the ECHA reports (ECHA, 2016; Alvarez et al., 2021), *B. thuringiensis* serovar *kurstaki* strain HD-1 is resistant to penicillin, ampicillin, and cephalothin. This corresponds with the beta-lactamase AMR genes identified in the samples, encoded by *bla*, *bla2*, and *blaIII,* with 12, 19, and 43 coding mutations respectively, but without structural disruptions of the corresponding ORFs (Supplementary Table S8). Three other AMR genes were found, encoding resistance to fosfomycin (*fosB/fosBx1*), streptothricin (*satA*), and vancomycin (*vanZ-F*), of which the former contained only a single coding mutation but the latter two exhibited truncations rendering their respective ORFs disrupted. According to EFSA and ECHA, this strain is susceptible to vancomycin, in agreement with the ORF of *satA* being disrupted, but no information was provided on fosfomycin and streptothricin. Since none of the isolates nor metagenomic data contained any unexpected AMR genes and only contained exactly the same genes as found in the reference, this further confirmed the authenticity of the samples. In terms of insecticidal genes, all isolates contained the same insecticidal genes as the reference, again down to the single nucleotide level. The only two exceptions were isolate 1 of sample 3, where a plasmid containing *cry1Ab3* was likely present at a very low copy number making it impossible to confirm the presence of the full gene, and isolate 2 of sample 4 which contained a chimeric insecticidal gene consisting partly of *cry1Ab3* and *cry1Ac5*. The chimeric nature of this gene was afterward independently confirmed through PCR amplification and Sanger sequencing of the gene in the isolate, highlighting the potential of WGS to detect genomic alterations as compared to targeted methods. The chimeric construct could not be found through PCR amplification and Sanger sequencing of the DNA extracted directly from the sample, indicating that this construct was only present in very few bacteria. Both the chimeric *cry* gene and the plasmid present at a very low copy number can most likely be explained as being a result of natural events. *B. thuringiensis* is known to spontaneously lose plasmids that contain *cry* genes (González et al., 1981) and experience recombination events (Shikov et al., 2023). Since the safety evaluation by EFSA did not specify which alleles of which insecticidal genes should be present in this bacterial strain, we had to rely on publicly available reference genome information for *B. thuringiensis* serovar *kurstaki* strain HD-1. Additional investigation against the literature confirmed the expected *cry* genes in the reference genome (see Supplementary Text S2). The results of our analyses therefore indicated that all samples contain the labeled strain and could consequently be considered as being authentic.

Potential contaminants from other species, or even from the same species through the presence of other strains of *B. thuringiensis*, can potentially be missed with isolate sequencing. This could for instance be the case if the contaminant was present in an untested colony, not viable under the growth conditions applied and therefore could not have been cultured, or alternatively because the contaminant was present at low relative abundances and therefore missed by the culturing of isolates. An open metagenomics approach was employed to sequence all DNA directly from the sample, using long-read sequencing to allow for a better characterization by allowing more complete genomes to be assembled (Loman et al., 2015). As long-read sequencing still suffers from an elevated error rate, short-read sequencing was also performed to reduce errors in the resulting hybrid assembly (Wick et al., 2021). Taxonomic classification with Kraken2 using a reference database containing a broad sampling from microorganisms indicated that only reads from the genus *Bacillus* were present. A follow-up investigation with StrainGE to perform strain-level analysis using a reference database containing all complete *Bacillus* genomes indicated that only genetic material from one specific cluster of extremely similar genomes that included *B. thuringiensis* serovar *kurstaki* strain HD-1 was present. All samples were hence found to be very pure. Due to the purity of the samples, the metagenomics data also allowed to independently evaluate the authenticity of samples. AMR and insecticidal gene content were exactly the same as in the reference and previously sequenced isolates, and SNP-based analysis indicated that all the samples were virtually identical to each other. Lastly, as the long reads allow to create much longer scaffolds, an analysis of potential large-scale structural variations that cannot always be detected with SNP-based approaches was performed. This showed that there were some small structural variations, likely the results of small-scale natural events, between the hybrid assemblies and the reference, but no large-scale differences that would indicate a different strain was present.

Since the analyzed products were found to be pure, a potential limitation of our study is that our methodology was not tested on a larger number of products, including more complex and contaminated products; as we only had access to four routine samples collected by the competent authorities from the European market. Future work should include a more diverse set of products, including impure and inauthentic ones, to extend the scope of the methodology and to get a better overview of the quality of different biopesticides on the market. We anticipate that the approach works on more complex samples because the analyses we performed are used in other fields of application on samples that often contain multiple species and/or strains. Kraken2 is often used for metagenomic read classification, for example in human gut microbiome analyses (Hiseni et al., 2021) and is capable of differentiating between a large number of species. StrainGE can differentiate between strains in complex clinical samples, even at low abundances (Lindstedt et al., 2022). These methods are hence expected to be able to verify the authenticity of biologically less complex samples such as microbial biopesticides.

With the rising popularity of microbial biopesticides as an alternative to traditional pesticides, it is important to be able to monitor the authenticity and purity of commercially available products, allowing to support the competent authorities in their control strategy. We proposed an approach that enables characterization of biopesticides using genomics technologies and assessed the added value of metagenomics and long-read sequencing. We tested four products containing *B. thuringiensis* serovar *kurstaki* strain HD-1 sold on the European market and found them to be authentic and pure, ensuring customers get the desired product and no biological contaminants are spread in the environment. Our findings suggest that the commercial bioinsecticides we have tested are of high quality, even though a limited set of samples were analyzed. Our approach can be used to evaluate the authenticity and purity of other microbial biopesticides and similar products. Nanopore sequencing quality is consistently improving, and the introduction of the novel oxford nanopore technologies R10 flow cells is expected to increase sequencing quality (Sereika et al., 2022), potentially even allowing in the future to perform detailed characterization without requiring additional short read sequencing and hybrid assembly strategies. Integration of this approach will aid in ensuring the overall safety of microbial biopesticides spread in the environment. It could also be useful for post-market surveillance of commercial samples by the competent authorities. A limiting factor is the confidentiality of the reference sequence. For the strain analyzed in this study, a suitable alternative was publicly available. Consequently, making the reference genome sequence public for biopesticides would enable more efficient control by the competent authorities, and increase the trust of the population in these products.

## Data Availability

The datasets presented in this study can be found in online repositories. The names of the repository/repositories and accession number(s) can be found at: https://www.ncbi.nlm.nih.gov/bioproject/PRJNA1085300. The Kraken2 database used for taxonomic classification of the metagenomic sequencing data is available for download on Zenodo (doi: 10.5281/zenodo.14589478).
