# Supplementary material for: Assessing the authenticity and purity of a commercial Bacillus thuringiensis bioinsecticide through whole genome sequencing and metagenomics approaches
Source: Front Microbiol. 2025 Jan 31;16:1532788. doi: 10.3389/fmicb.2025.1532788 (PMC11831548; doi:10.3389/fmicb.2025.1532788)
Supplement: Supplementary file 1 [file Data_Sheet_1.docx]

# Figures

## Figure S1: Visualization of the total DNA extracted from the samples


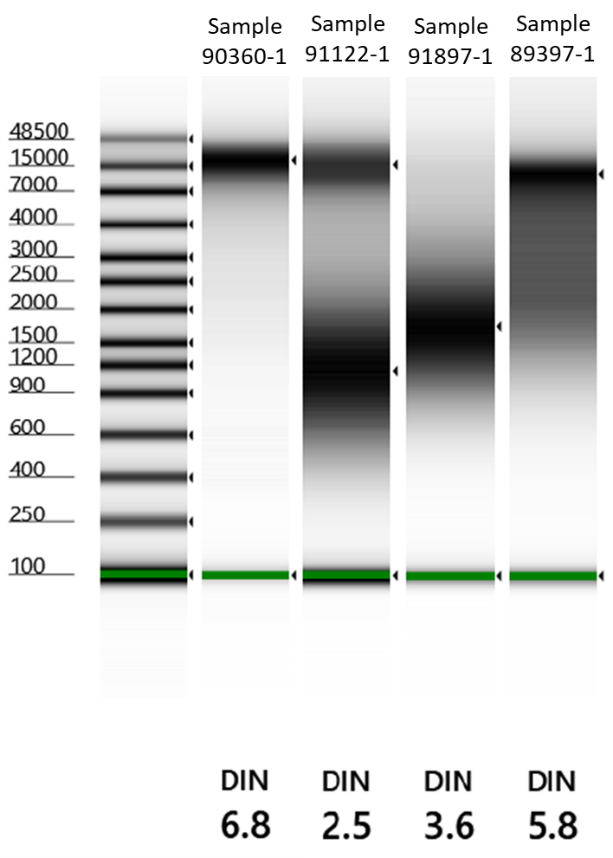


## Figure S2: Visualization of the PCR products


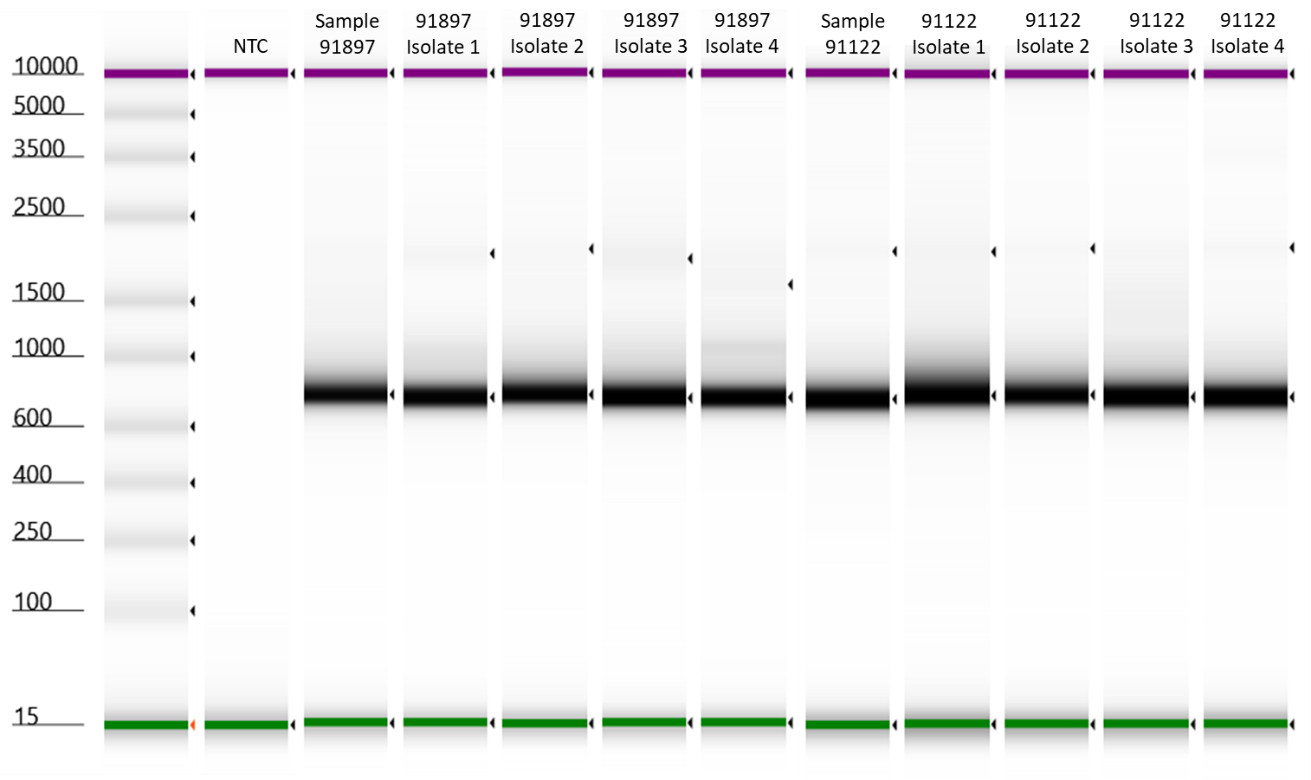


## Figure S3: Read mapping results for the isolates of sample 1 at the location of the *cry1Aa8* gene


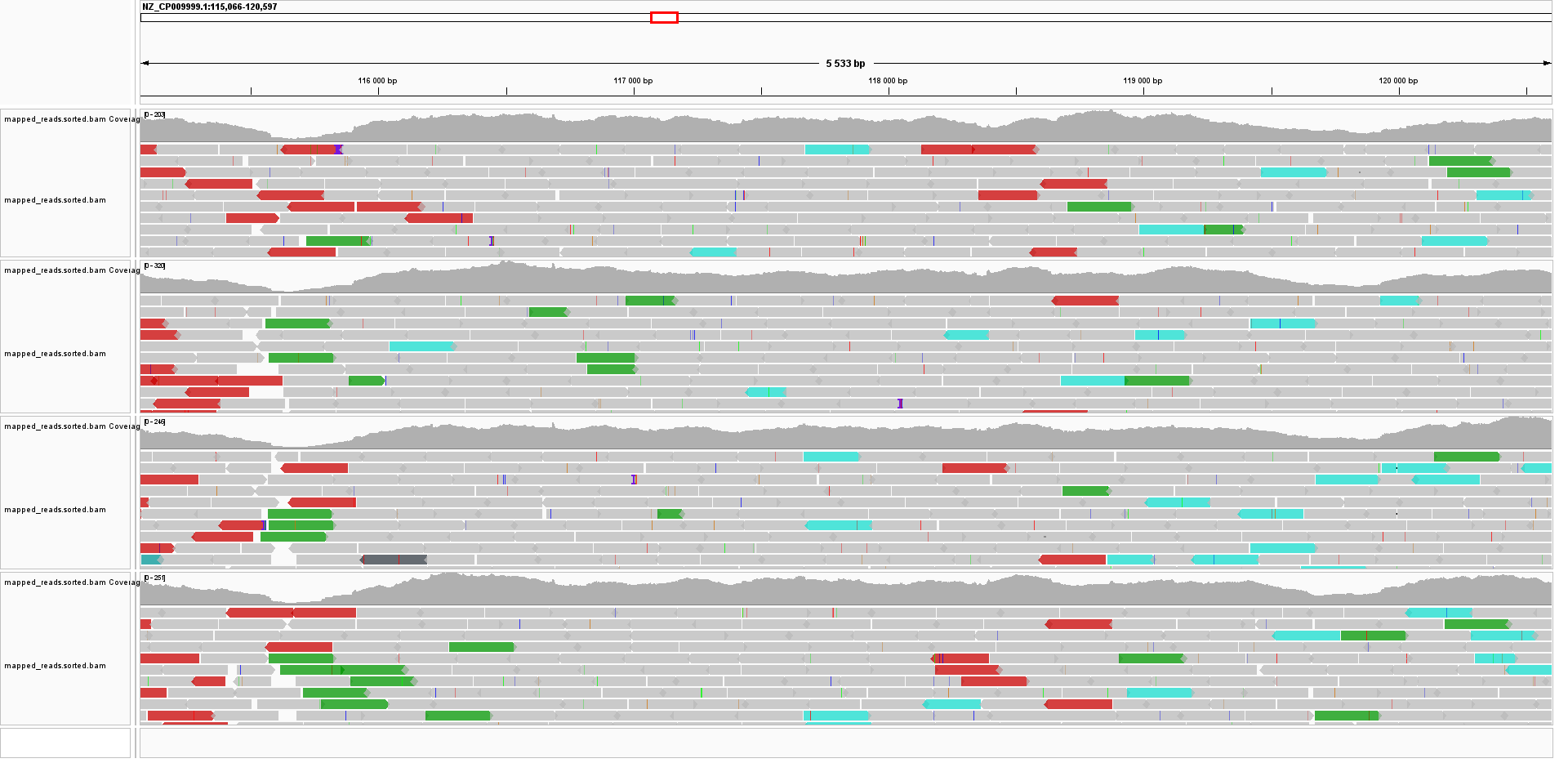


The observed window contains the *cry1Aa8* gene with 1000 bp surroundings on both sides. From top to bottom, the results of isolates 1-4 are shown.

## Figure S4: Read mapping results for the isolates of sample 1 at the location of the *cry1Ab3* gene


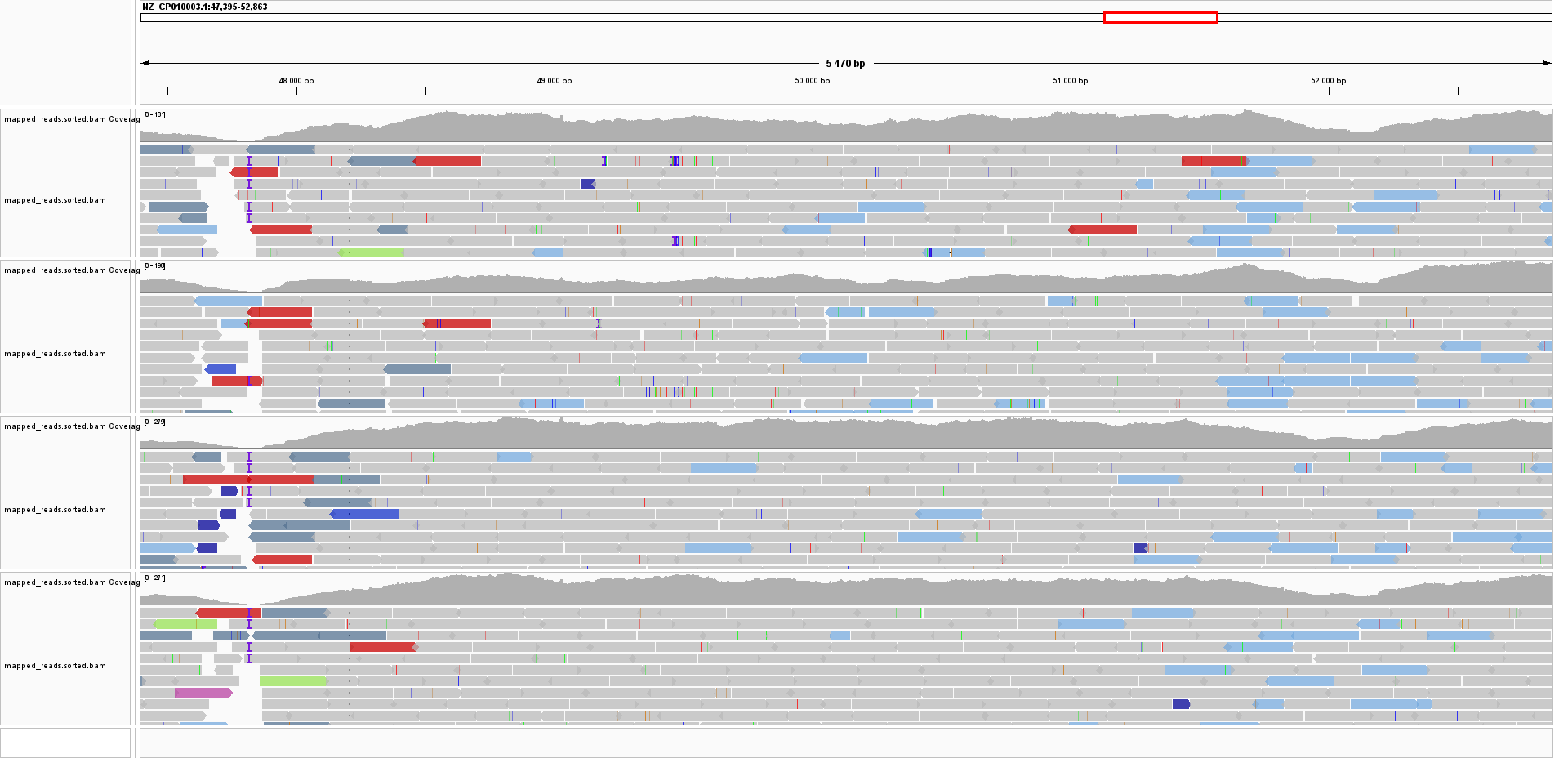


The observed window contains the *cry1Ab3* gene with 1000 bp surroundings on both sides. From top to bottom, the results of isolates 1-4 are shown.

## Figure S5: Read mapping results for the isolates of sample 1 at the location of the *cry1Ac5* gene


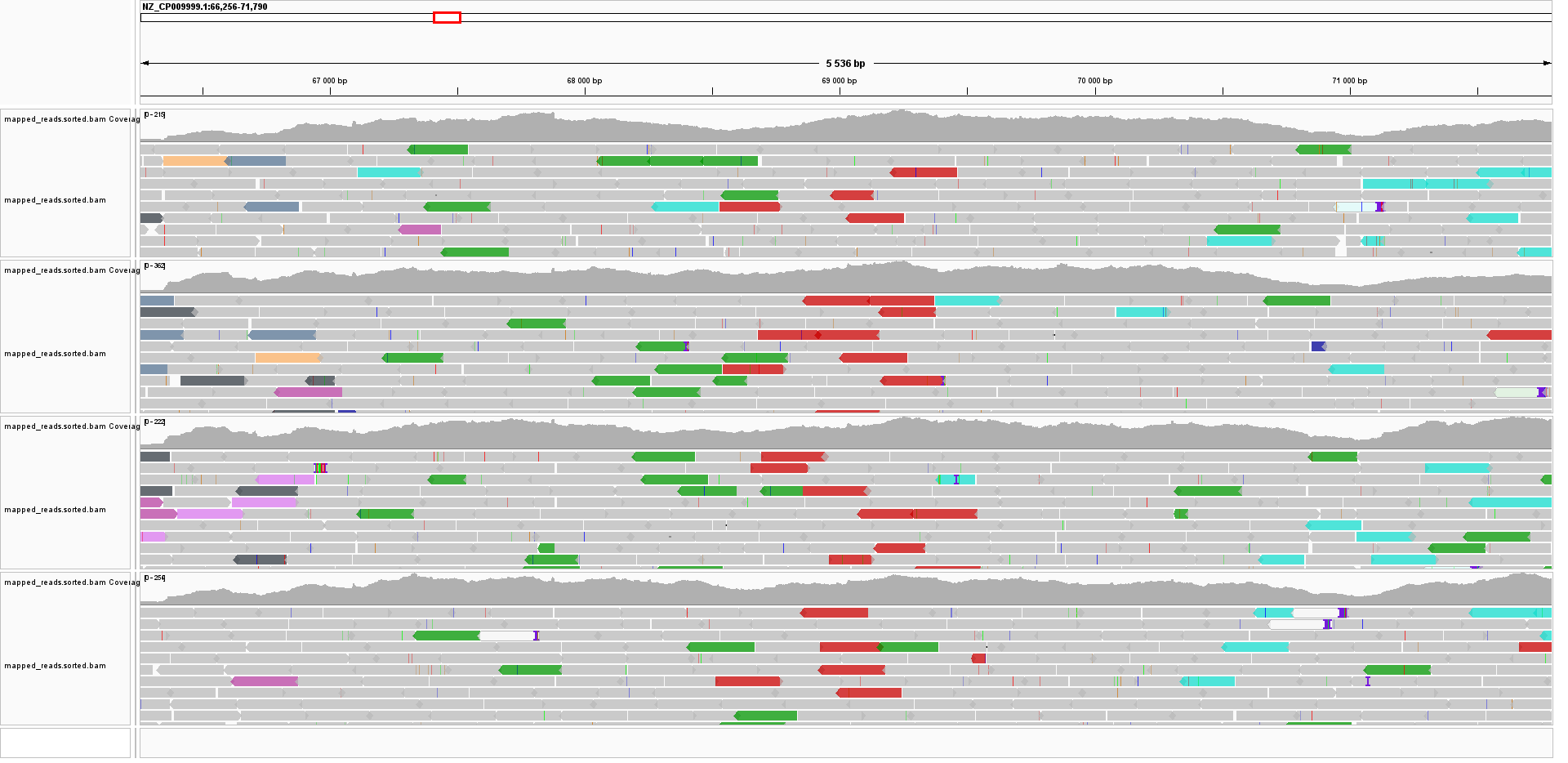


The observed window contains the *cry1Ac5* gene with 1000 bp surroundings on both sides. From top to bottom, the results of isolates 1-4 are shown.

## Figure S6: Read mapping results for the isolates of sample 2 at the location of the *cry1Aa8* gene


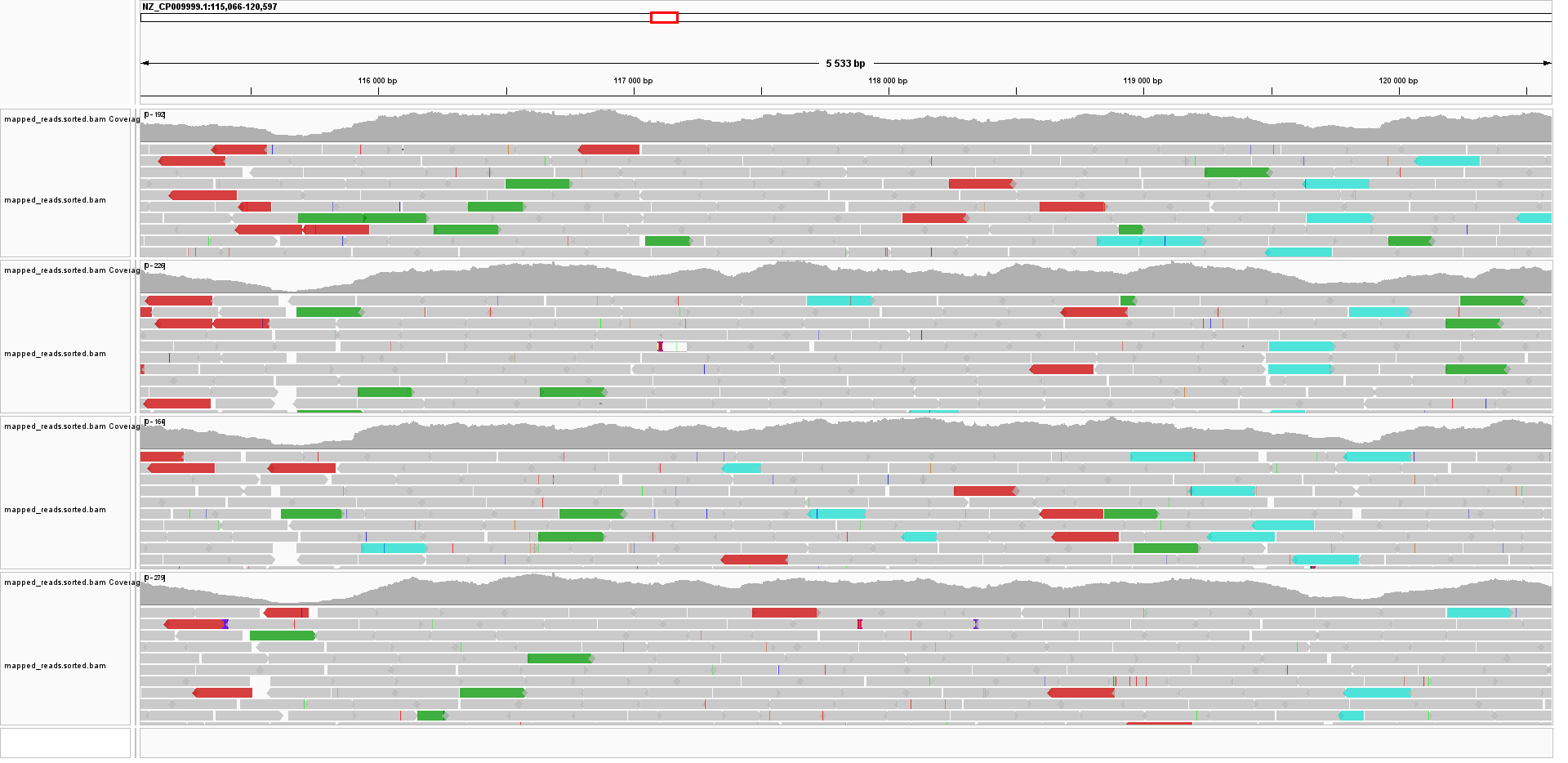


The observed window contains the *cry1Aa8* gene with 1000 bp surroundings on both sides. From top to bottom, the results of isolates 1-4 are shown.

## Figure S7: Read mapping results for the isolates of sample 2 at the location of the *cry1Ab3* gene


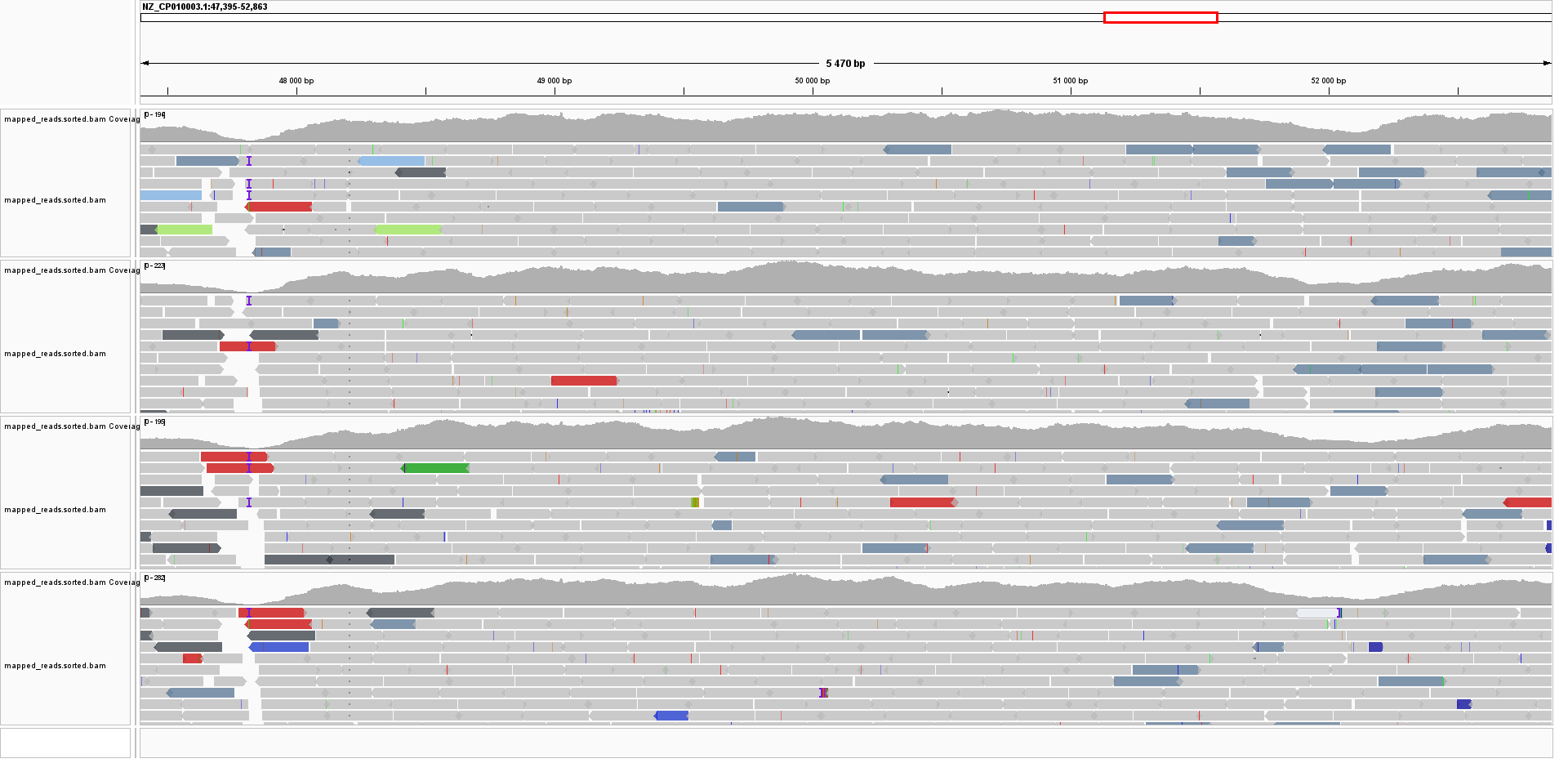


The observed window contains the *cry1Ab3* gene with 1000 bp surroundings on both sides. From top to bottom, the results of isolates 1-4 are shown.

## Figure S8: Read mapping results for the isolates of sample 2 at the location of the *cry1Ac5* gene


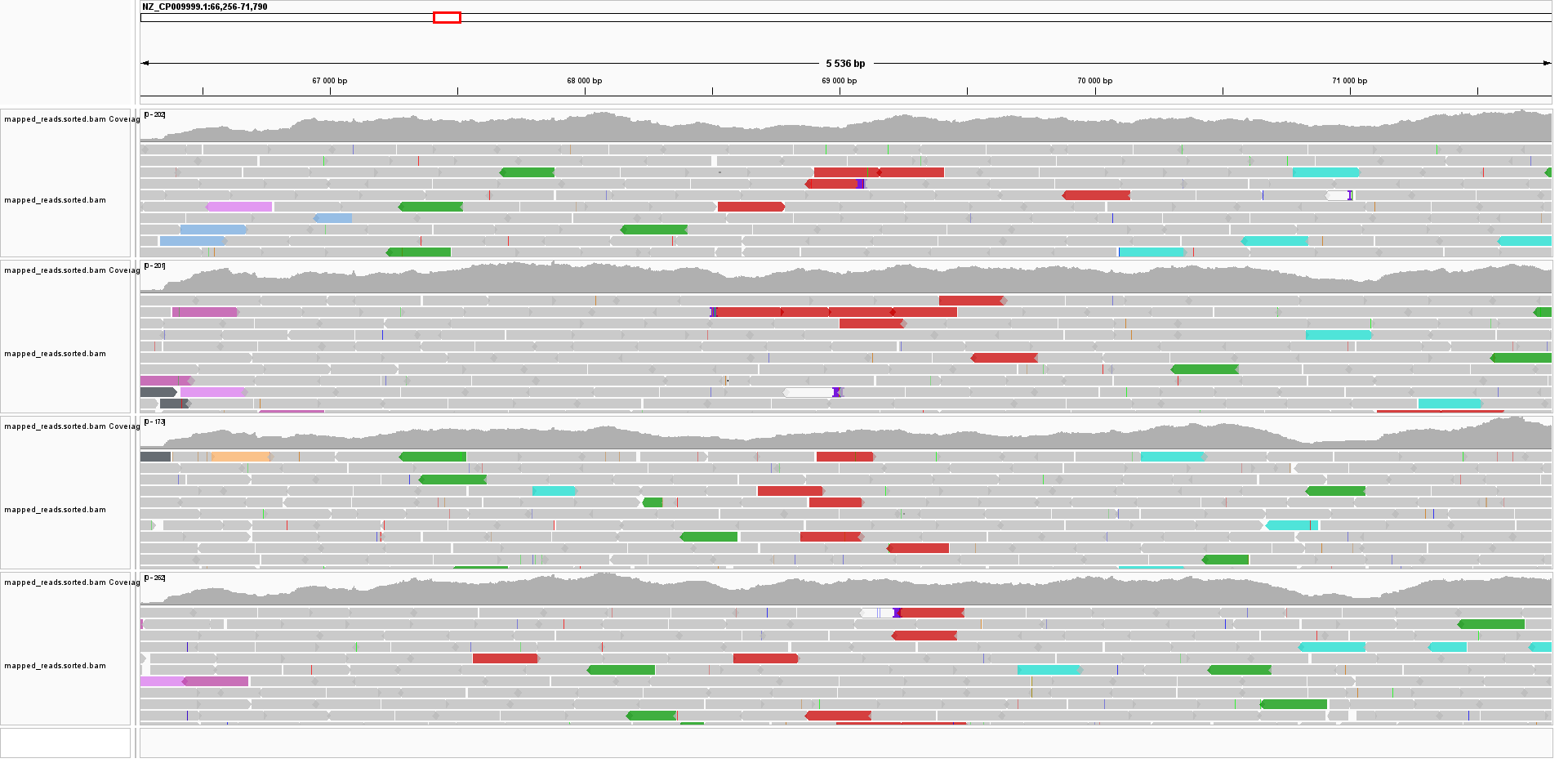


The observed window contains the *cry1Ac5* gene with 1000 bp surroundings on both sides. From top to bottom, the results of isolates 1-4 are shown.

## Figure S9: Read mapping results for the isolates of sample 3 at the location of the *cry1Aa8* gene


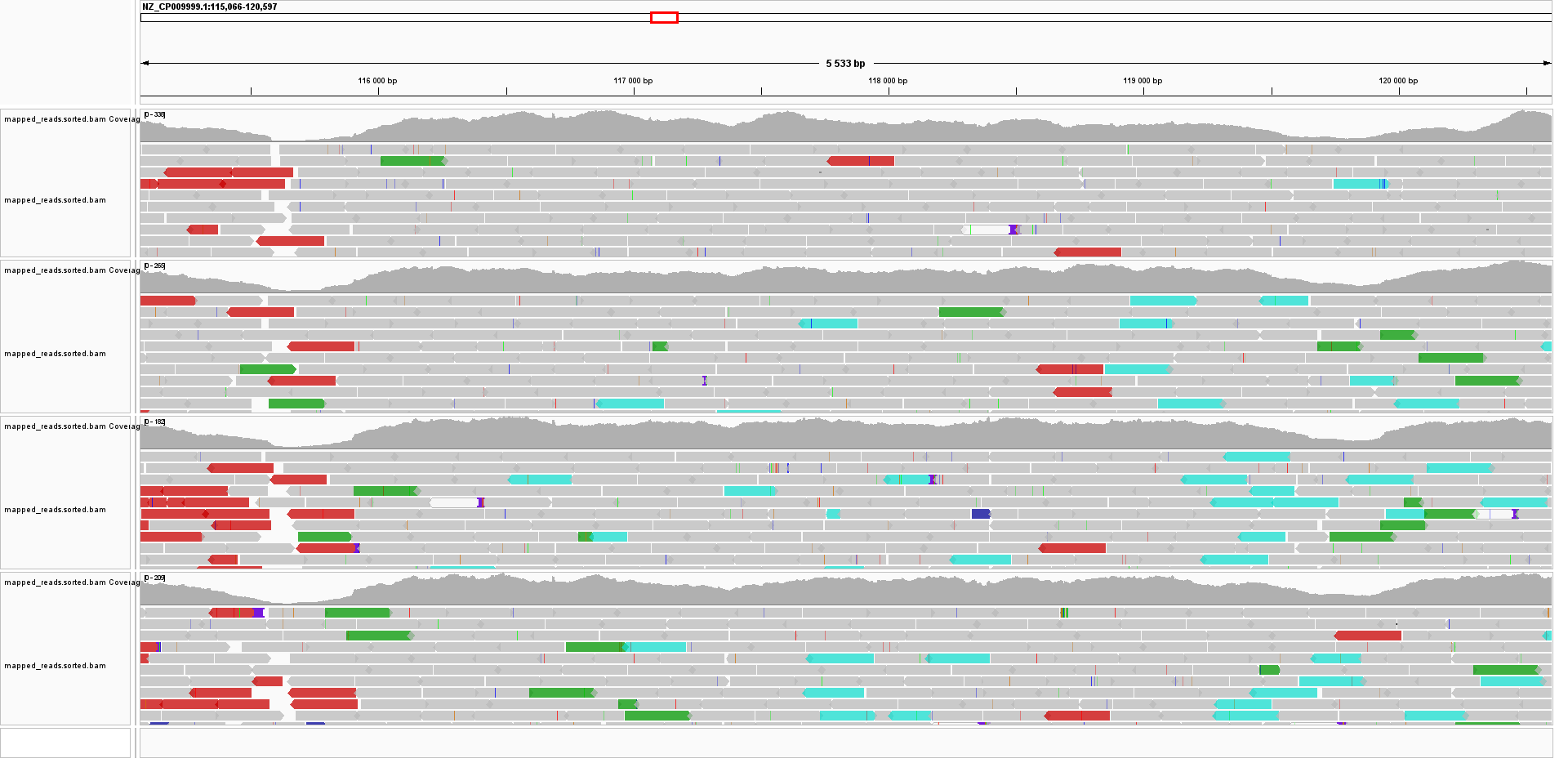


The observed window contains the *cry1Aa8* gene with 1000 bp surroundings on both sides. From top to bottom, the results of isolates 1-4 are shown.

## Figure S10: Read mapping results for the isolates of sample 3 at the location of the *cry1Ab3* gene


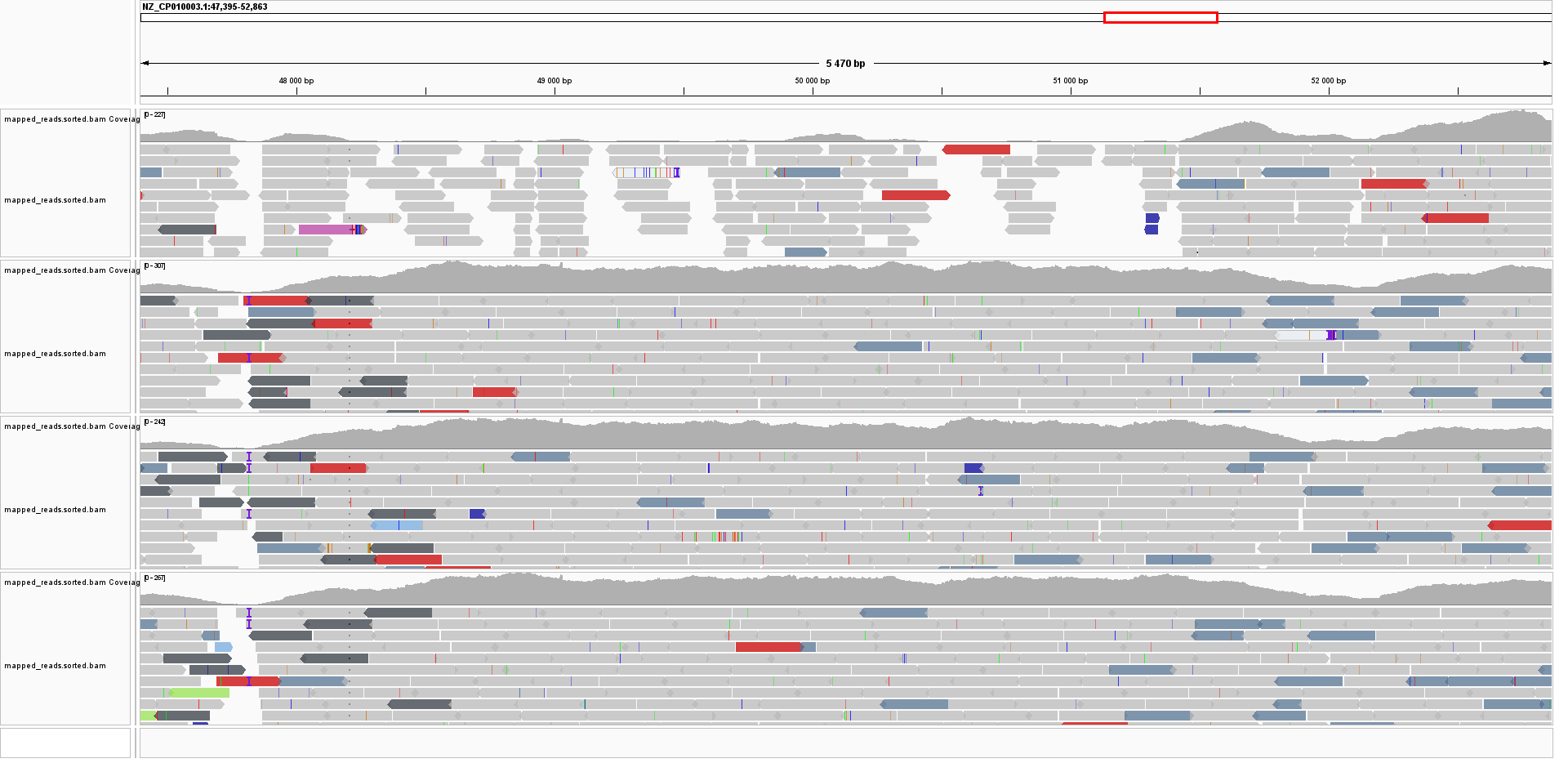


The observed window contains the *cry1Ab3* gene with 1000 bp surroundings on both sides. From top to bottom, the results of isolates 1-4 are shown. In isolate 1, the breadth of coverage for the plasmid containing the *cry1Ab3* gene is relatively low (92.57%). The mean mapq value is also low (6.39). Out of the 9 599 reads that map to the plasmid, 8 216 have a mapq of 1 and 1 145 have the maximum mapq value of 42. The remaining 238 reads have mapq values between 1 and 42. When only considering reads with the highest mapq value of 42, the breadth and depth of coverage for the plasmid were 58.07% and 3.25 respectively. This indicates that a considerable number of reads align uniquely with this plasmid, although the frequency is comparatively lower than observed for other plasmids. Out of these reads with the highest mapq value, 15 mapped to the *cry1Ab3* gene.

## Figure S11: Read mapping results for the isolates of sample 3 at the location of the *cry1Ac5* gene


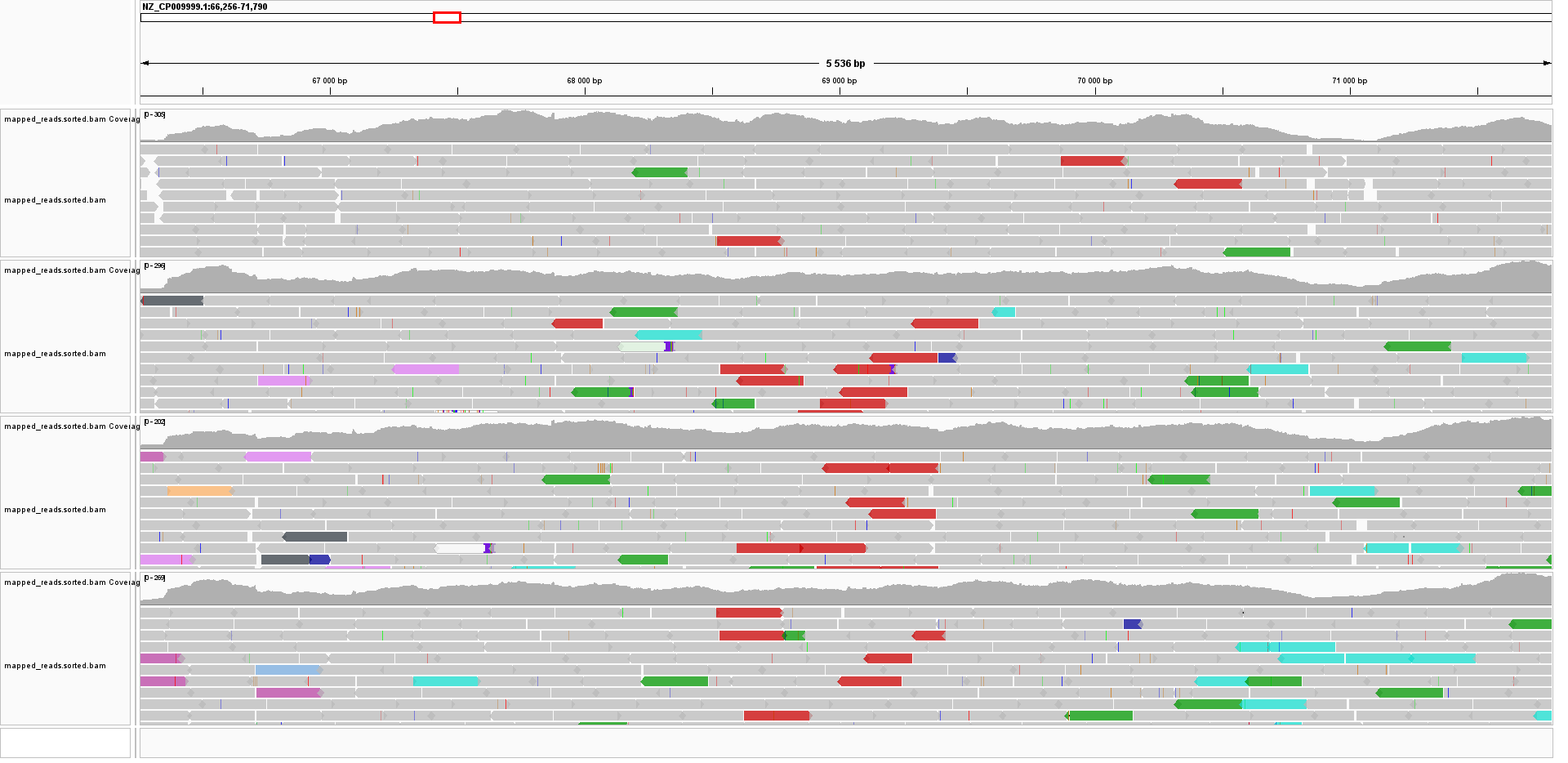


The observed window contains the *cry1Ac5* gene with 1000 bp surroundings on both sides. From top to bottom, the results of isolates 1-4 are shown.

## Figure S12: Read mapping results for the isolates of sample 4 at the location of the *cry1Aa8* gene


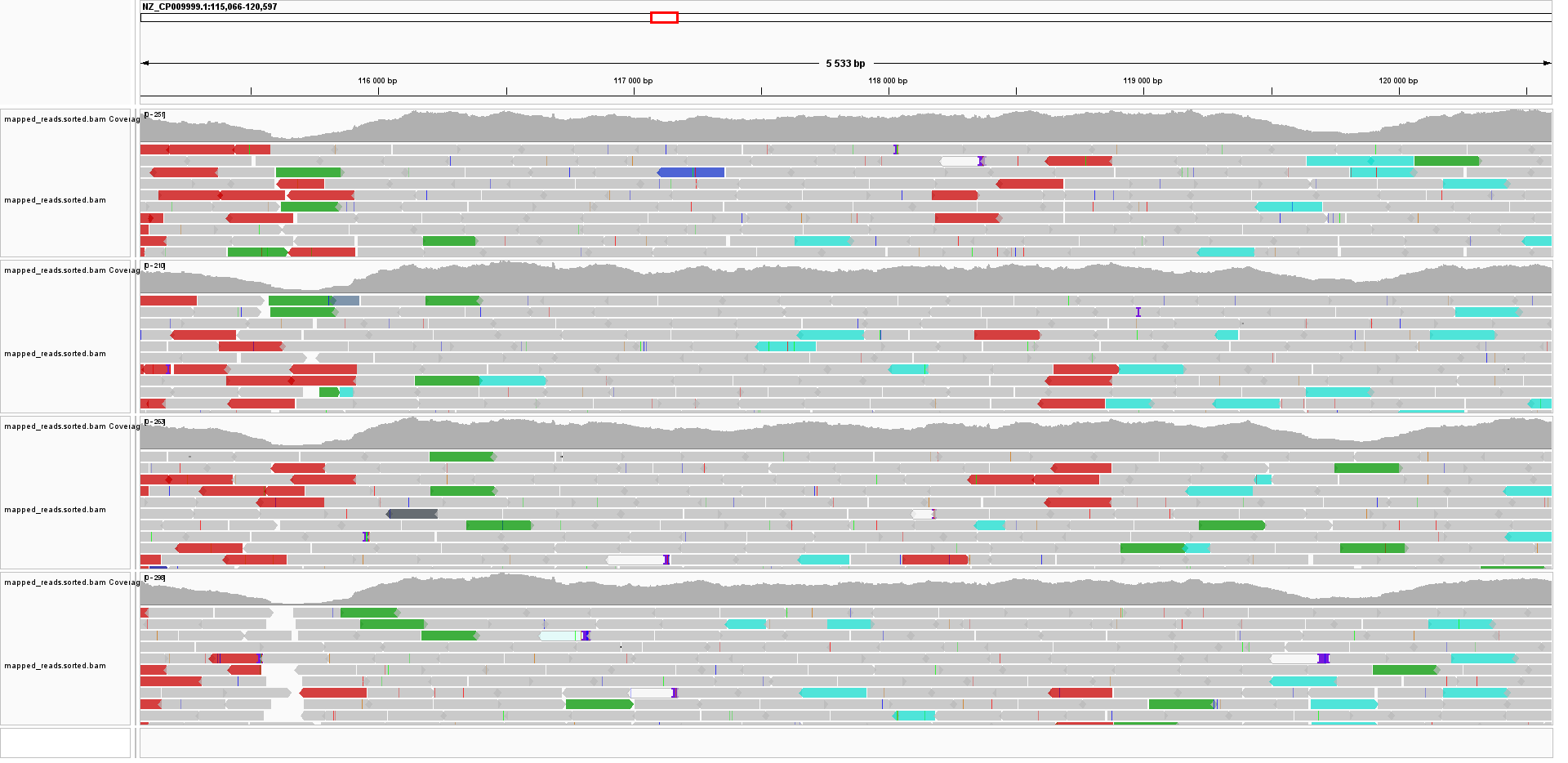


The observed window contains the *cry1Aa8* gene with 1000 bp surroundings on both sides. From top to bottom, the results of isolates 1-4 are shown.

## Figure S13: Read mapping results for the isolates of sample 4 at the location of the *cry1Ab3* gene


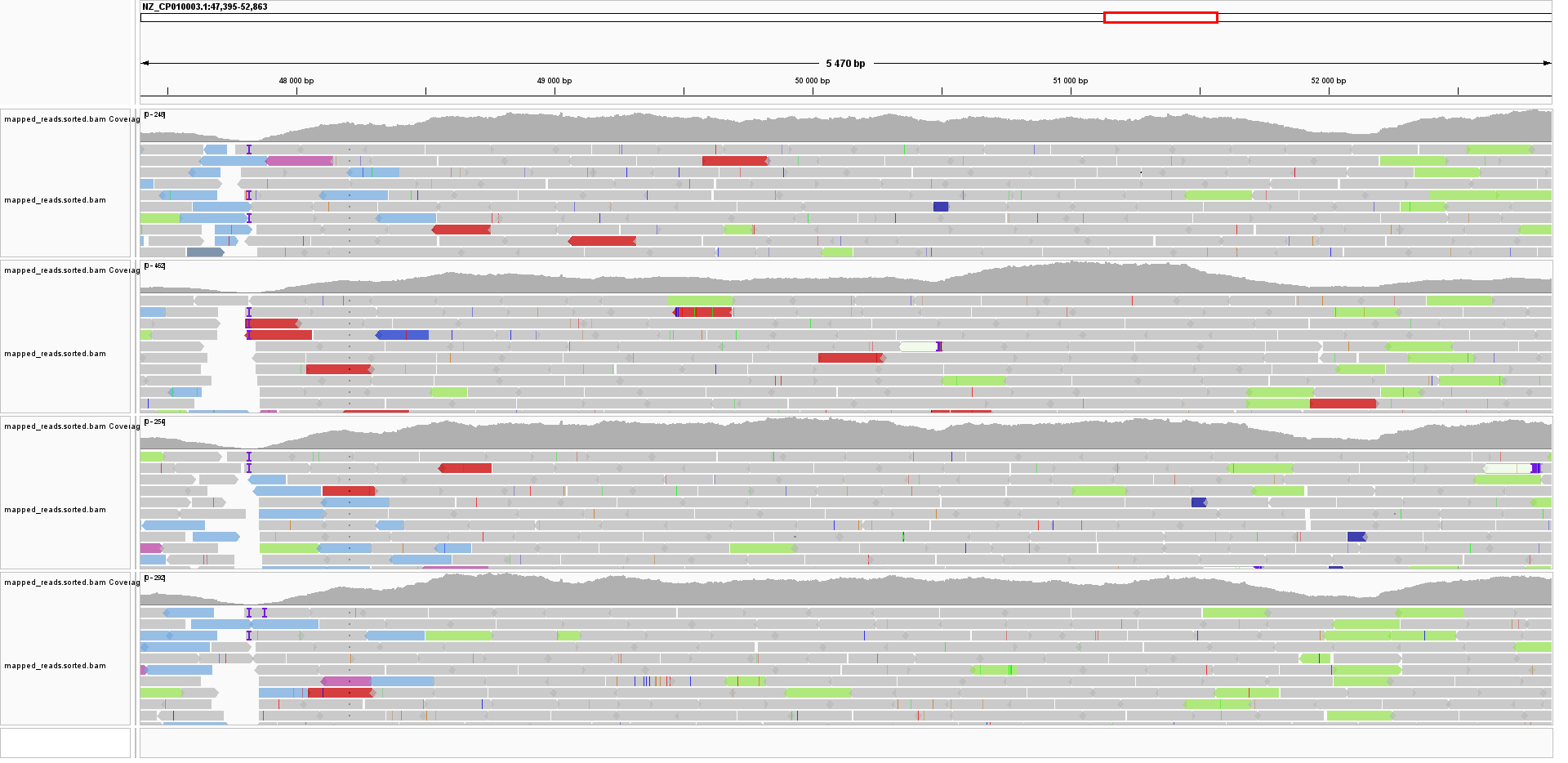


The observed window contains the *cry1Ab3* gene with 1000 bp surroundings on both sides. From top to bottom, the results of isolates 1-4 are shown.

## Figure S14: Read mapping results for the isolates of sample 4 at the location of the *cry1Ac5* gene


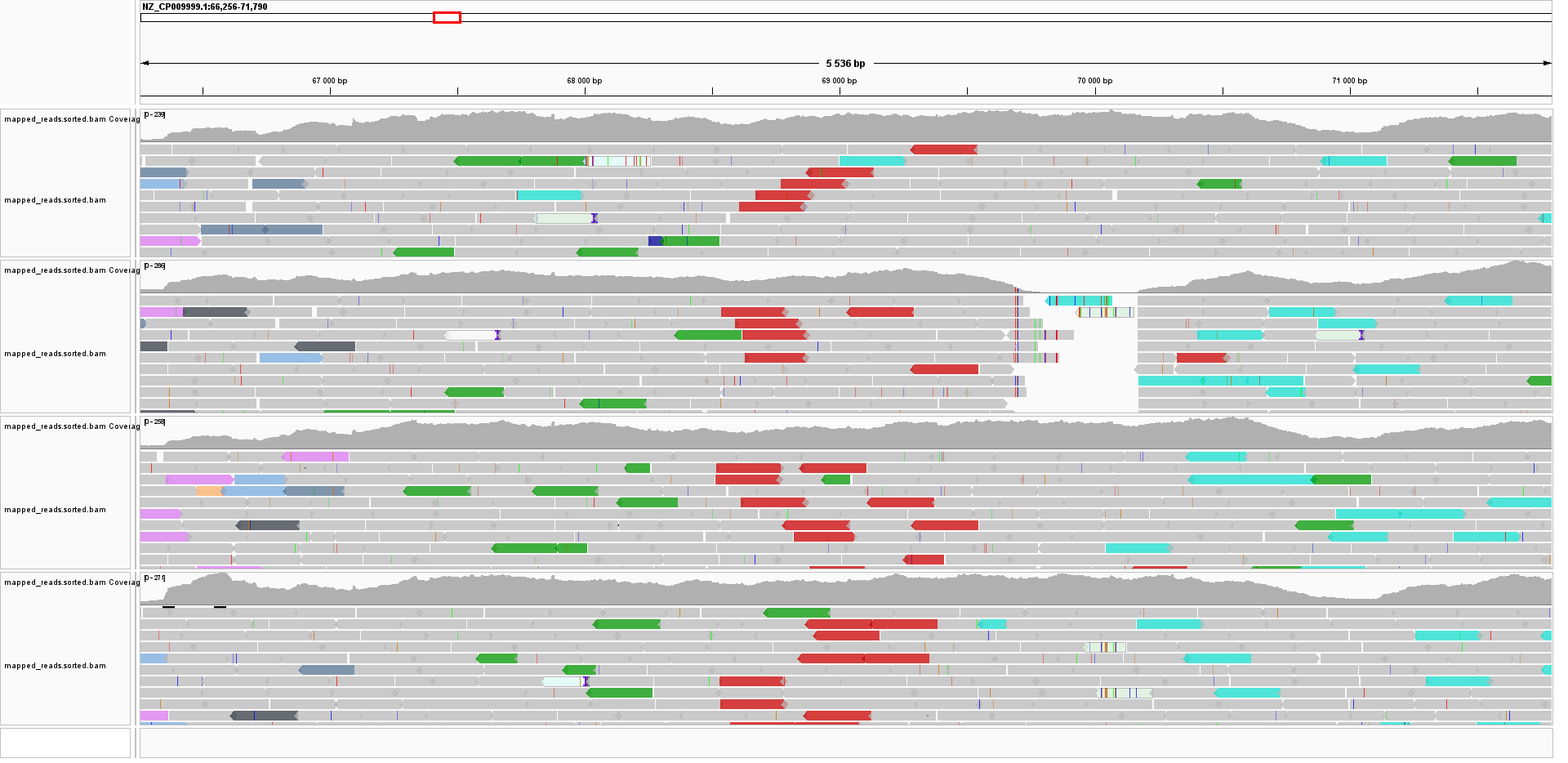


The observed window contains the *cry1Ac5* gene with 1000 bp surroundings on both sides. From top to bottom, the results of isolates 1-4 are shown.

## Figure S15: Krona visualization of the Kraken2 results of the short-read sequencing data of sample 1


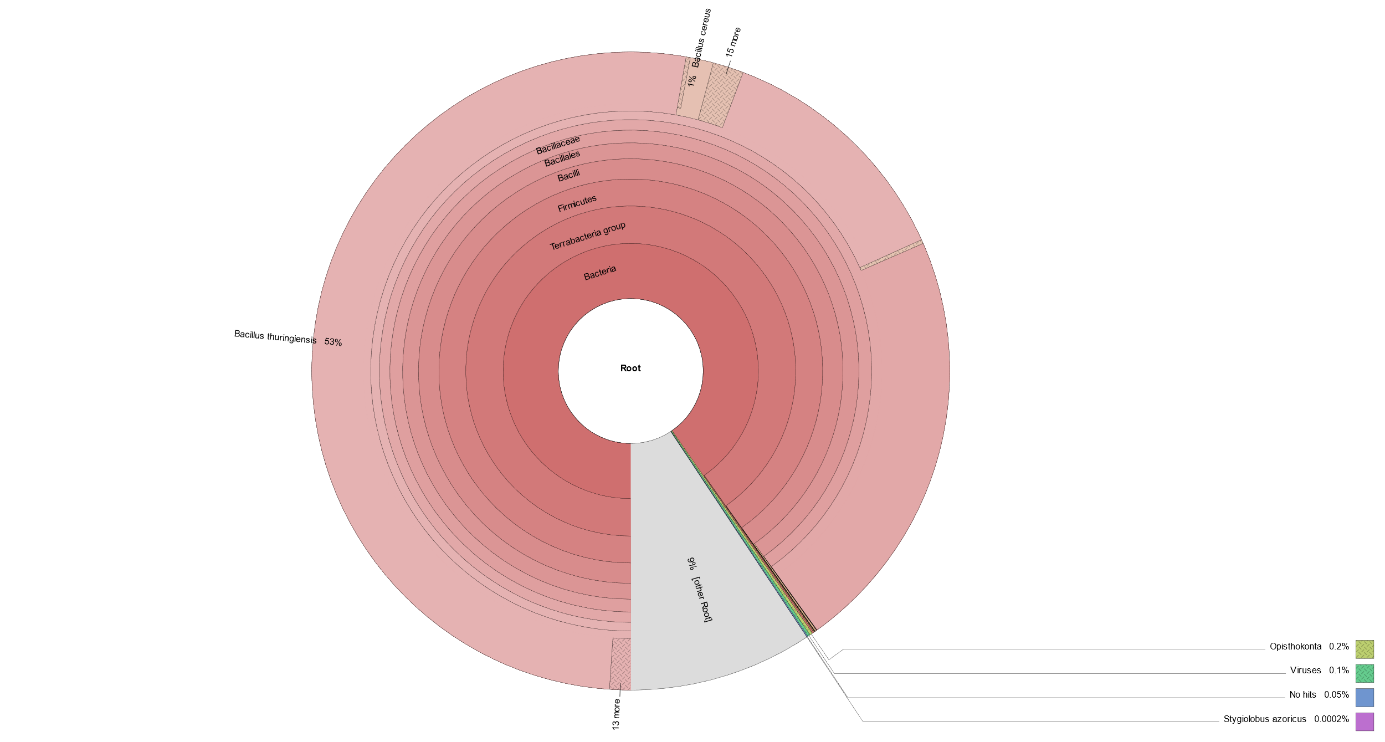


## Figure S16: Krona visualization of the Kraken2 results of the short-read sequencing data of sample 2


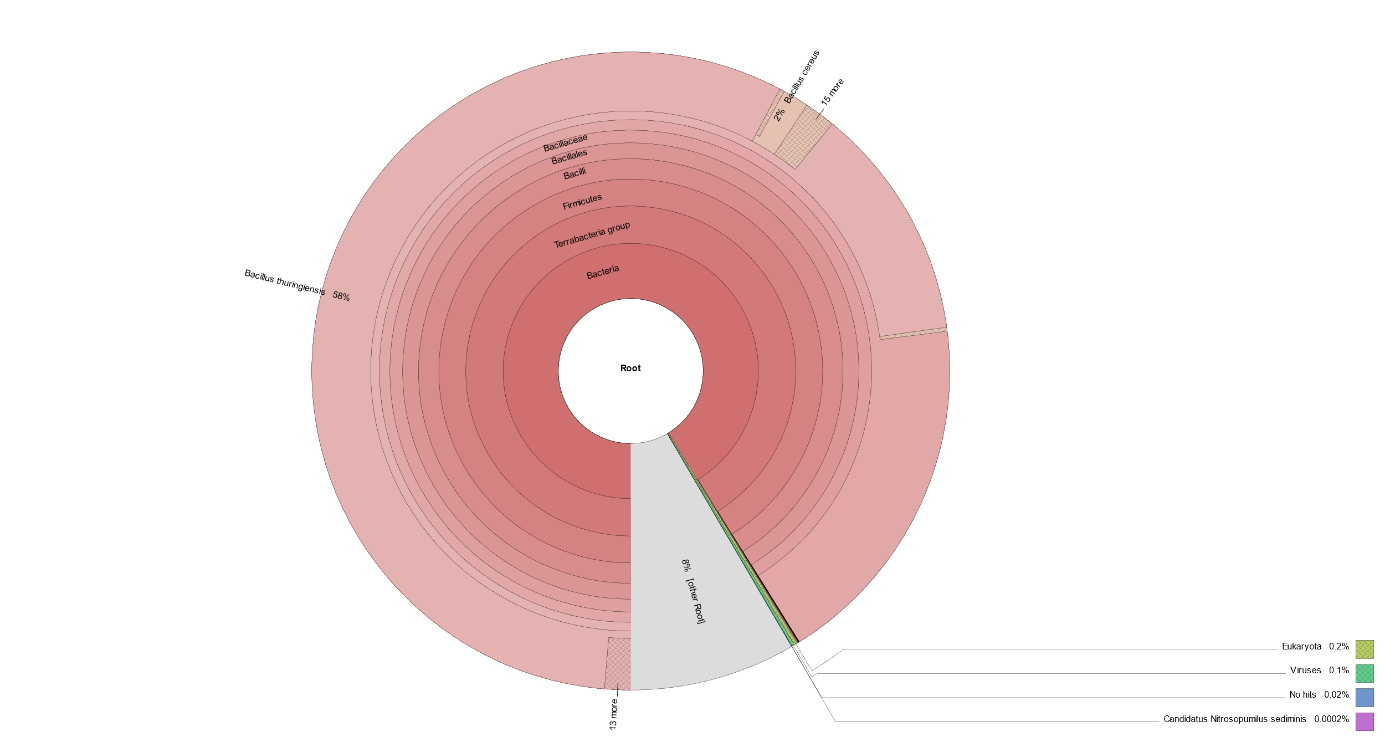


## Figure S17: Krona visualization of the Kraken2 results of the short-read sequencing data of sample 3


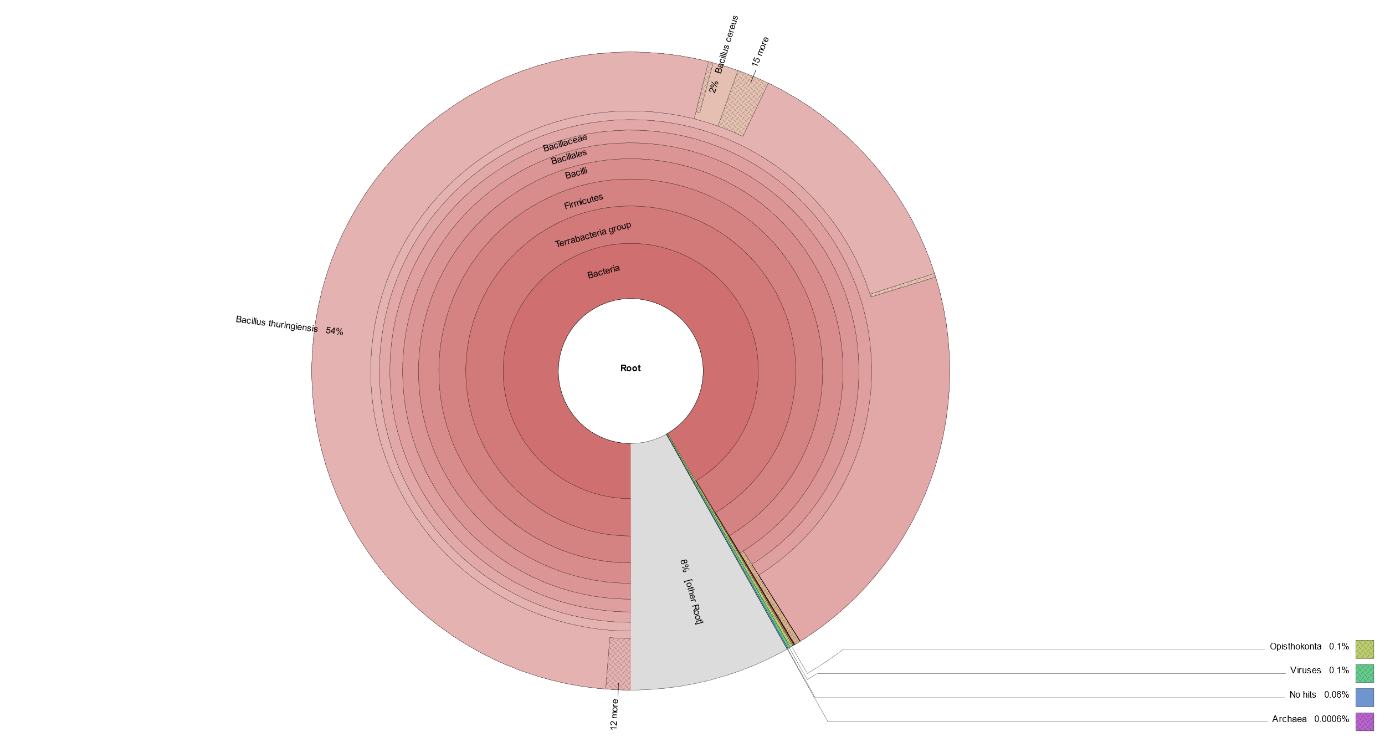


## Figure S18: Krona visualization of the Kraken2 results of the short-read sequencing data of sample 4


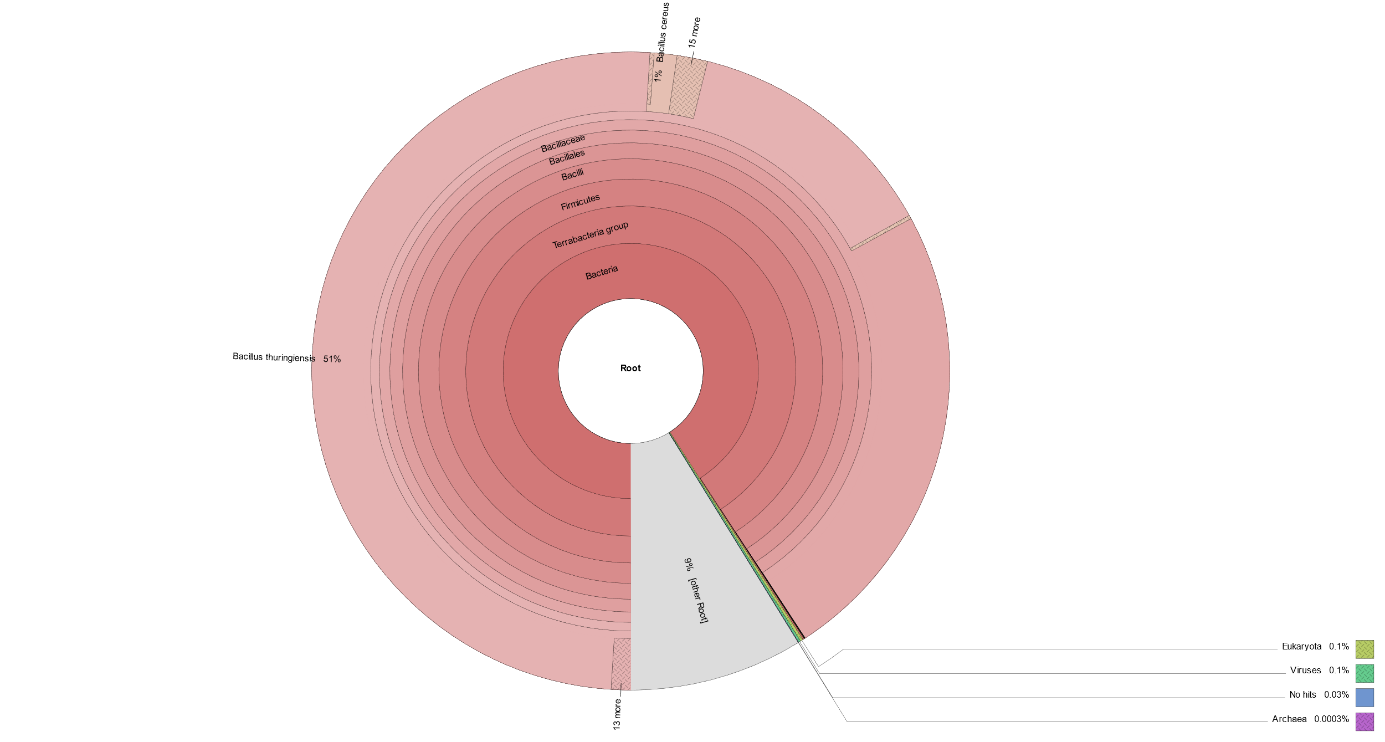


## Figure S19: Krona visualization of the Kraken2 results of the long-read sequencing data of sample 1


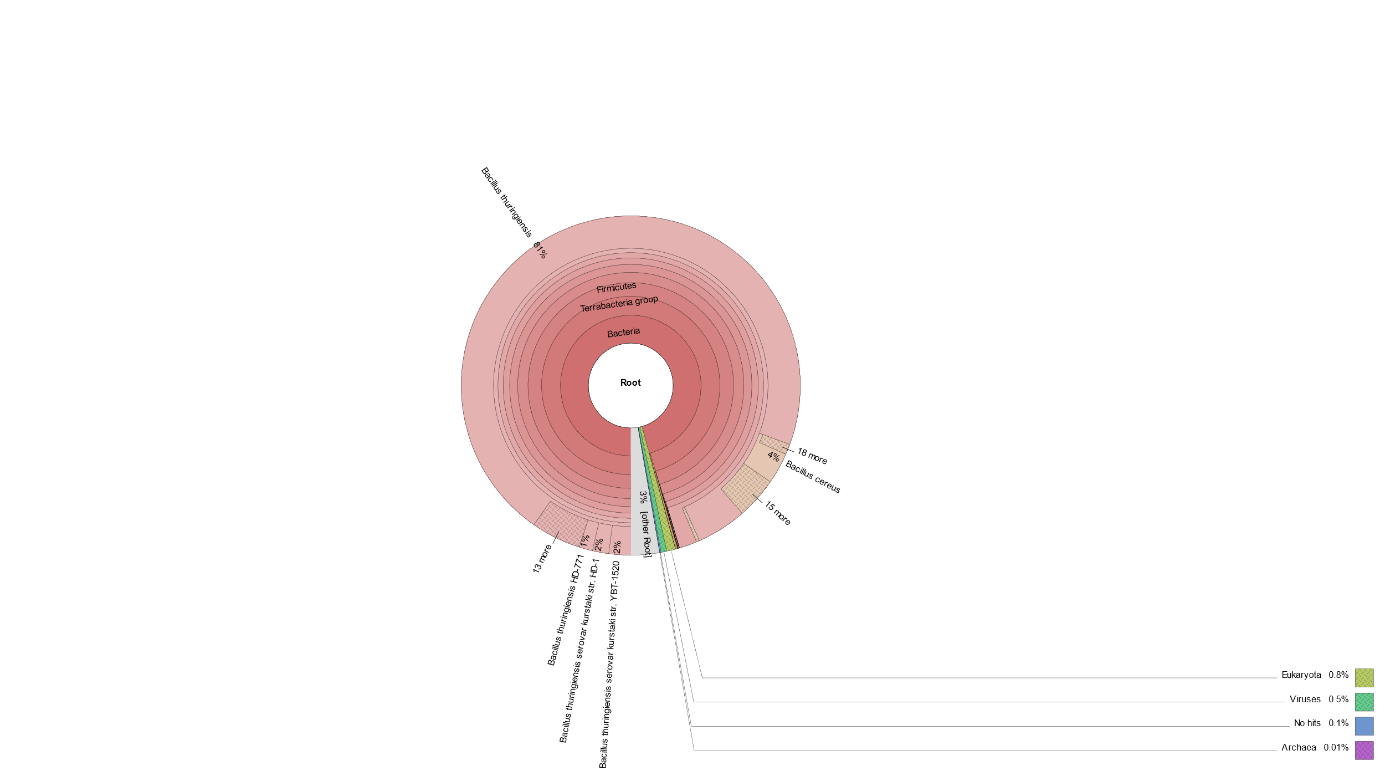


## Figure S20: Krona visualization of the Kraken2 results of the long-read sequencing data of sample 2


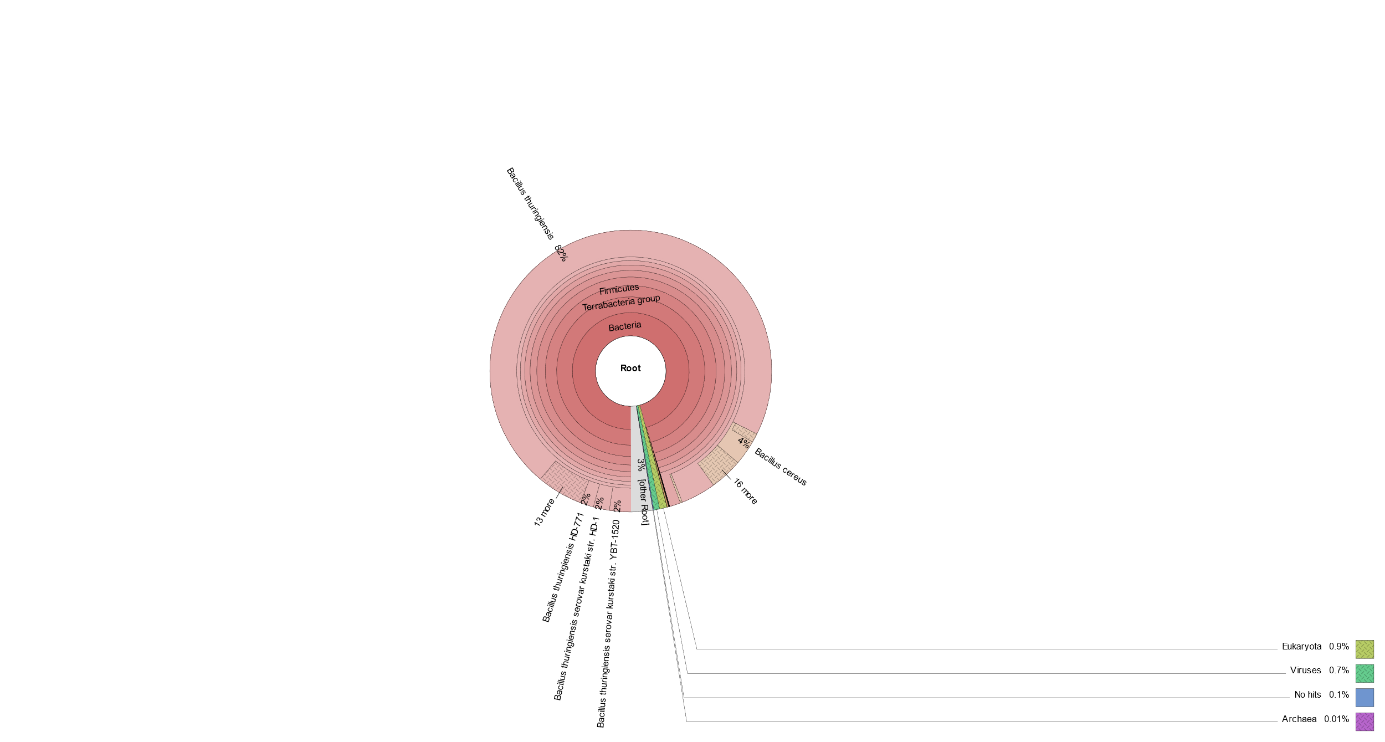


## Figure S21: Krona visualization of the Kraken2 results of the long-read sequencing data of sample 3


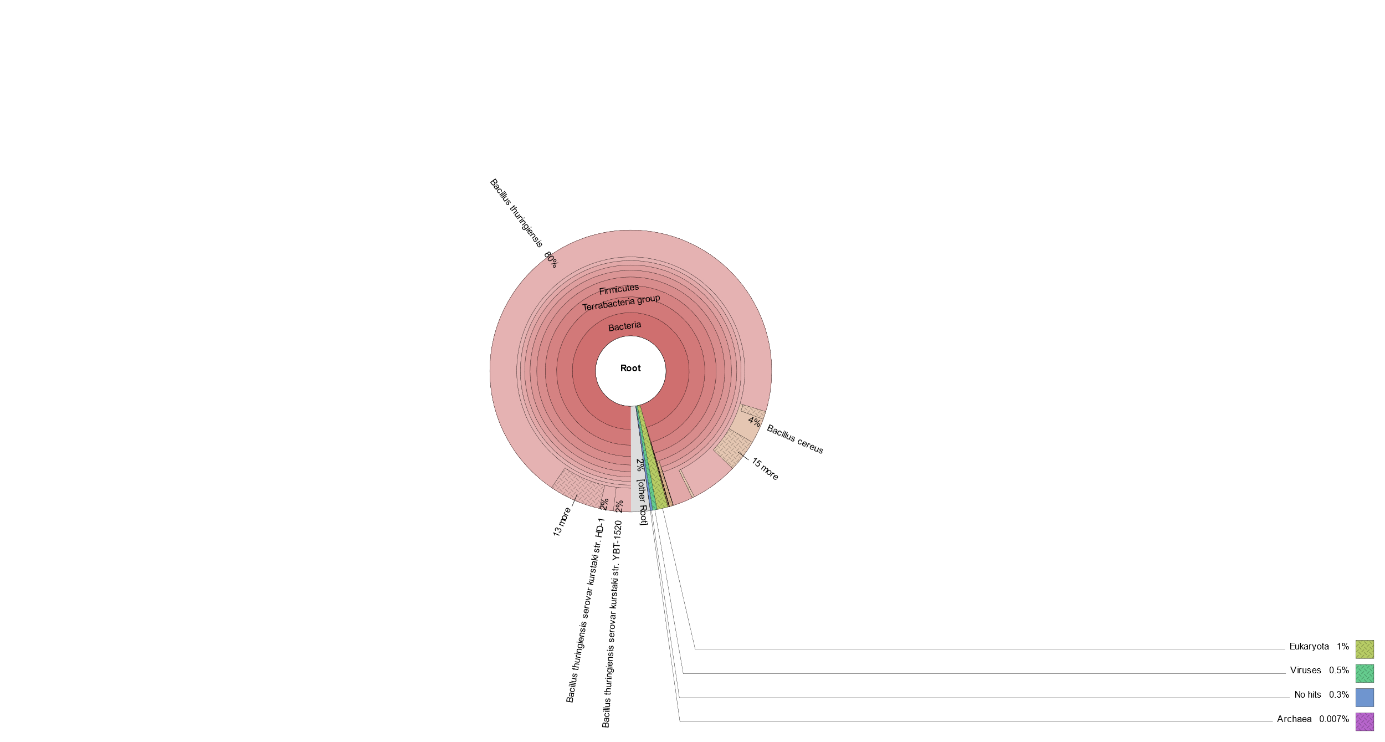


## Figure S22: Krona visualization of the Kraken2 results of the long-read sequencing data of sample 4


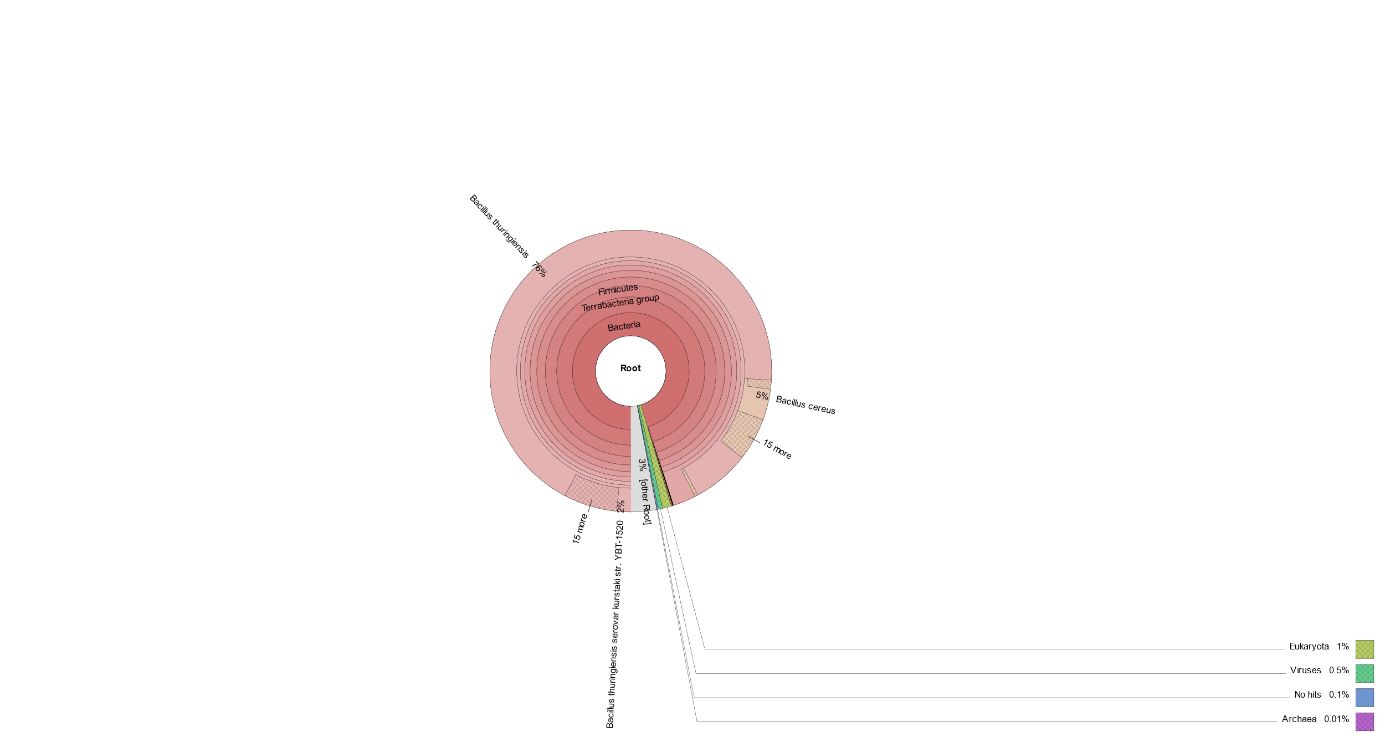


## Figure S23: Mummerplot to assess large-scale structural variation in sample 1


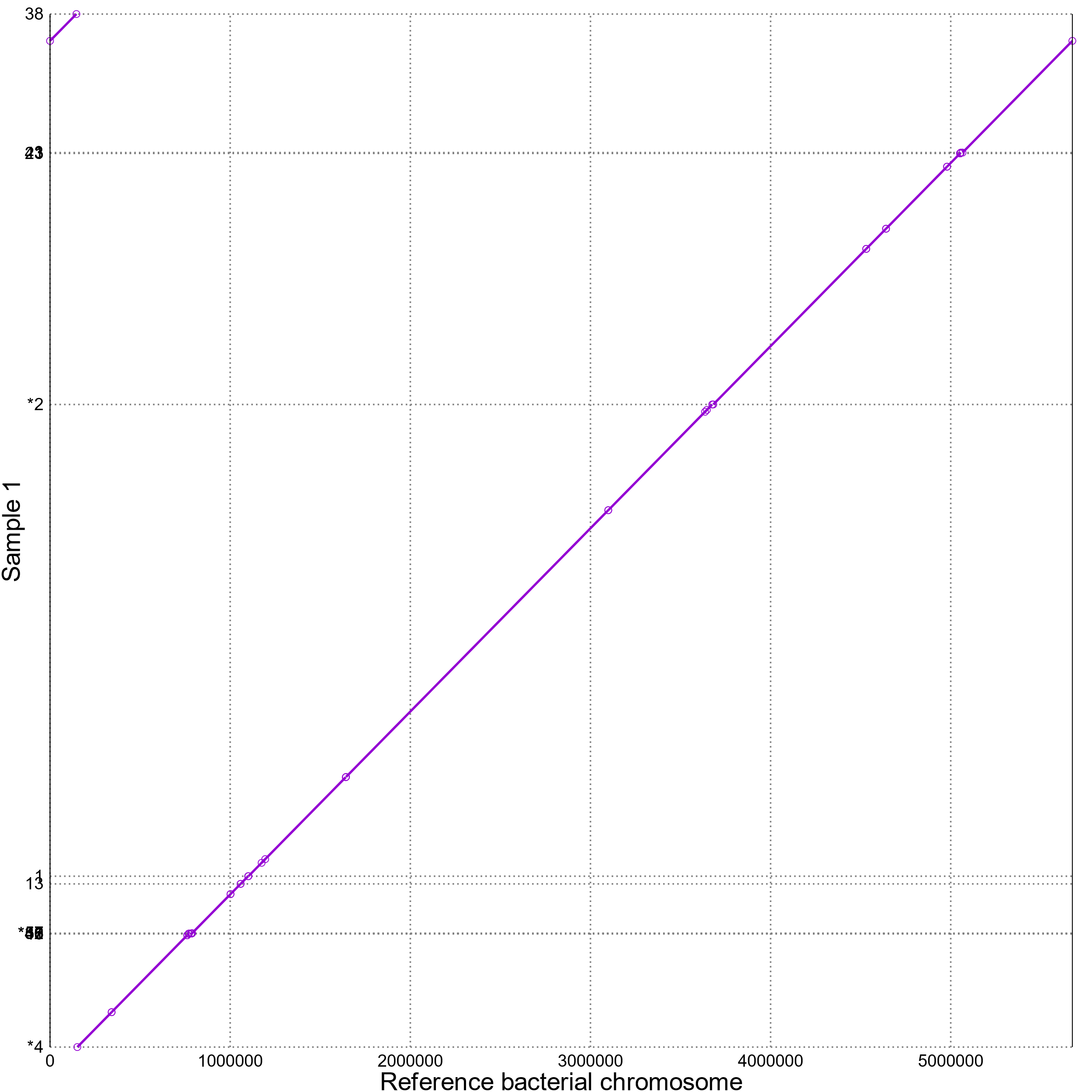


The y-axis shows the Unicycler assembly of sample 1, the labels correspond to contig numbers. The x-axis shows the bacterial chromosome of the reference genome, the labels correspond to genomic locations. The lines show regions of homology between the two. Purple lines indicate homology in the same direction, and blue lines indicate homology with the reverse complement. The circles indicate the presence of structural variations.

## Figure S24: Mummerplot to assess large-scale structural variation in sample 3


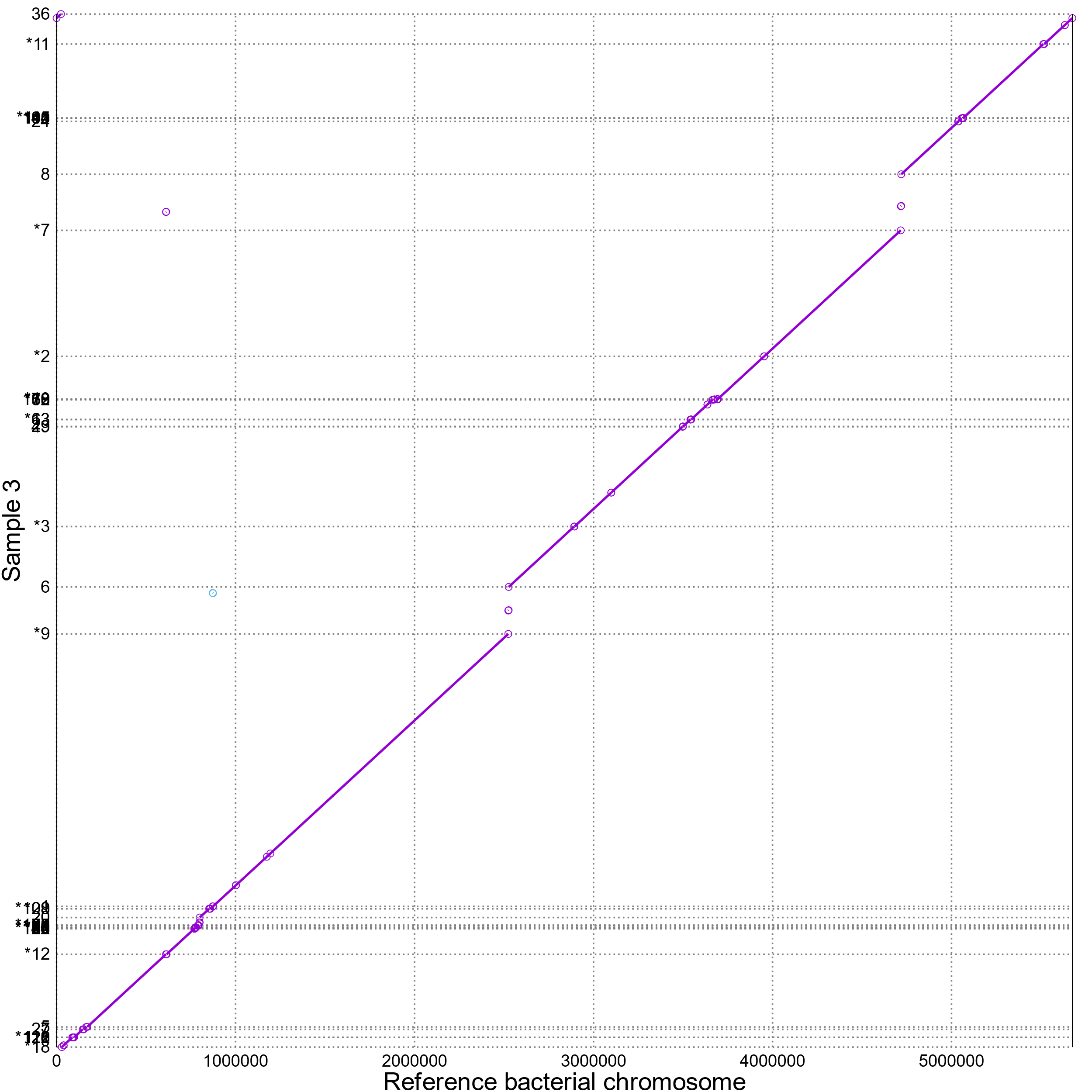


The y-axis shows the Unicycler assembly of sample 3, the labels correspond to contig numbers. The x-axis shows the bacterial chromosome of the reference genome, the labels correspond to genomic locations. The lines show regions of homology between the two. Purple lines indicate homology in the same direction, and blue lines indicate homology with the reverse complement. The circles indicate the presence of structural variations.

## Figure S25: Mummerplot to assess large-scale structural variation in sample 4


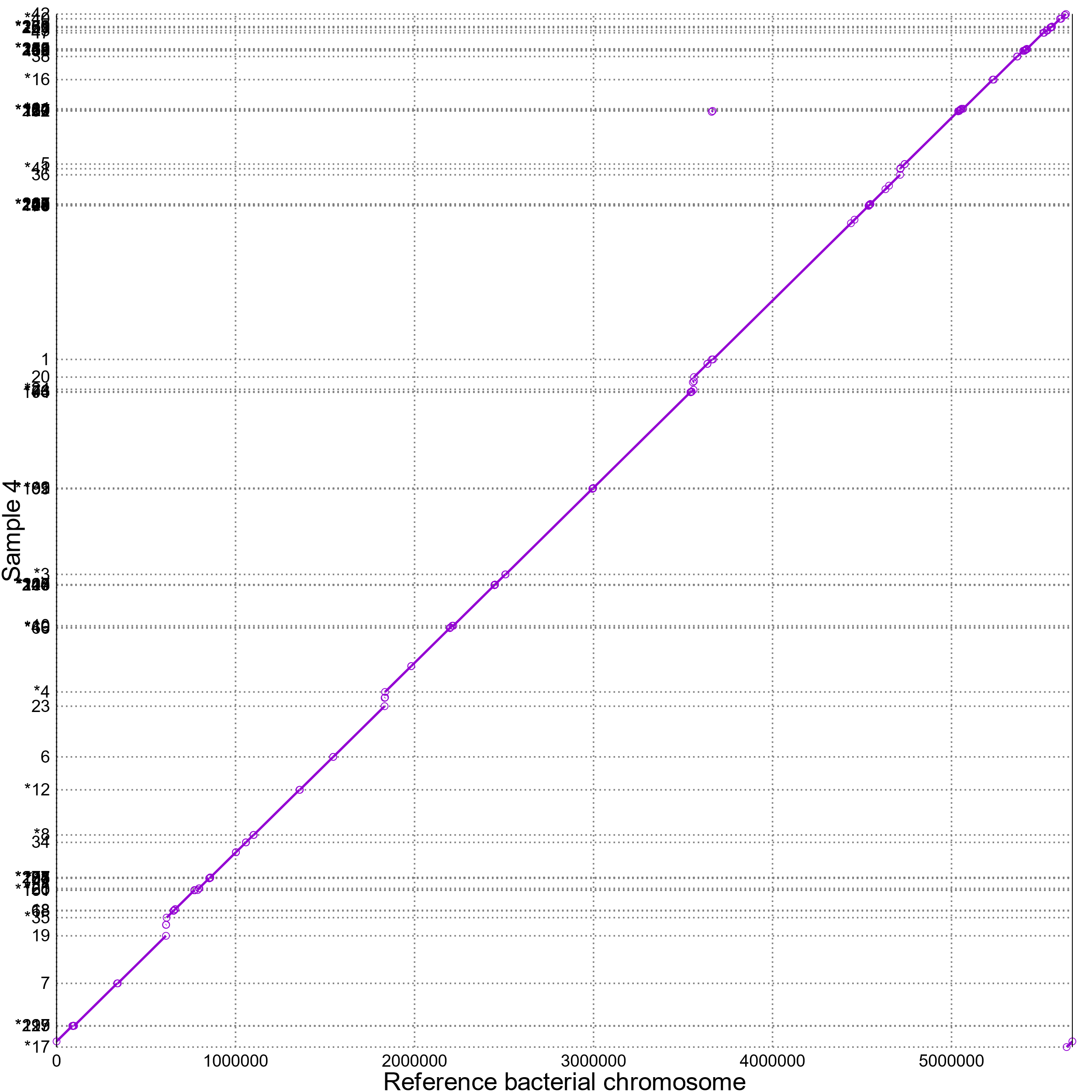


The y-axis shows the Unicycler assembly of sample 3, the labels correspond to contig numbers. The x-axis shows the bacterial chromosome of the reference genome, the labels correspond to genomic locations. The lines show regions of homology between the two. Purple lines indicate homology in the same direction, and blue lines indicate homology with the reverse complement. The circles indicate the presence of structural variations

## Figure S26: Read mapping results for the metagenomic short reads at the location of the *cry1Aa8* gene.


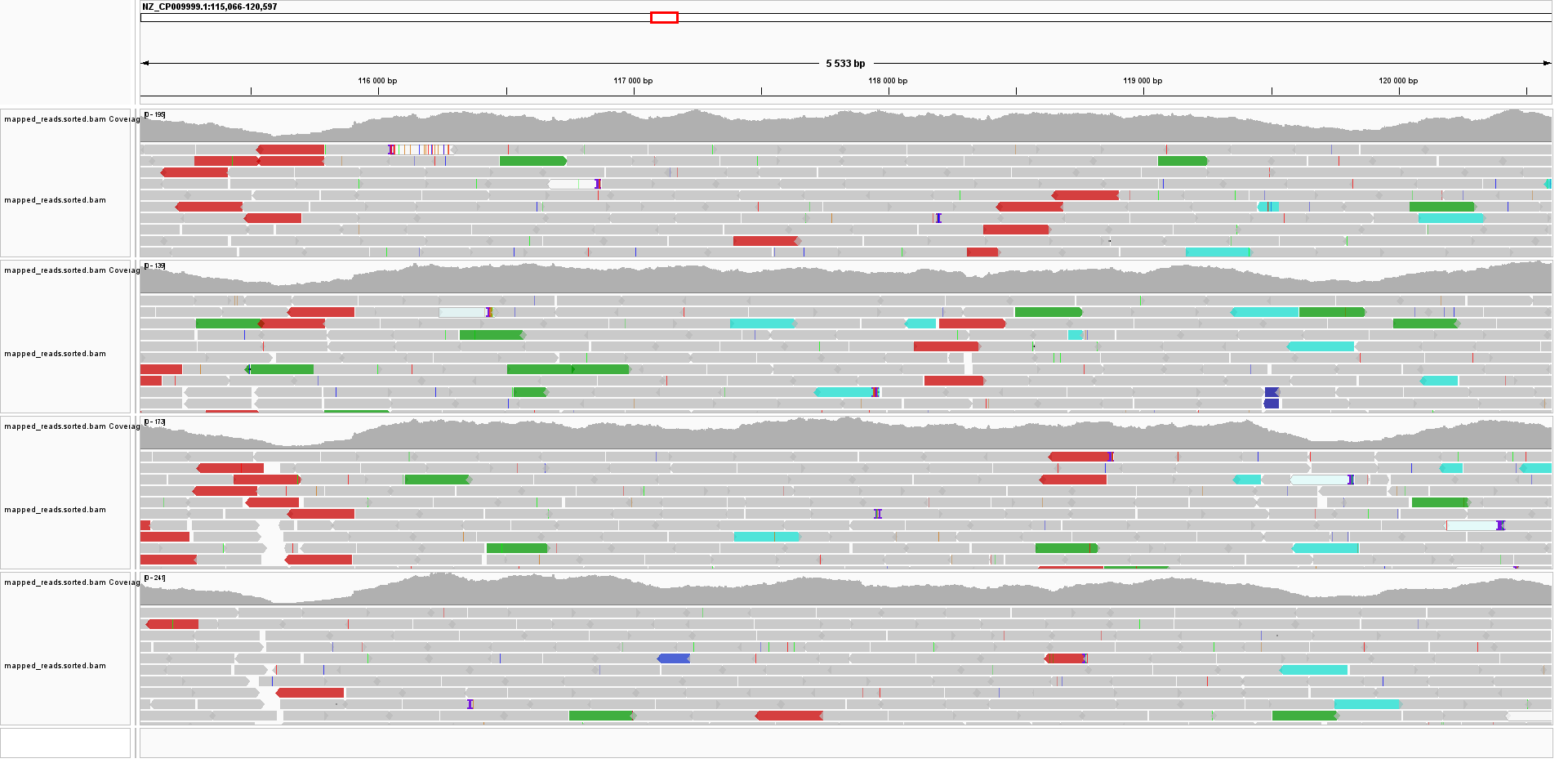


The observed window contains the *cry1Aa8* gene with 1000 bp surroundings on both sides. From top to bottom, the results of samples 1-4 are shown.

## Figure S27: Read mapping results for the metagenomic short reads at the location of the *cry1Ab3* gene.


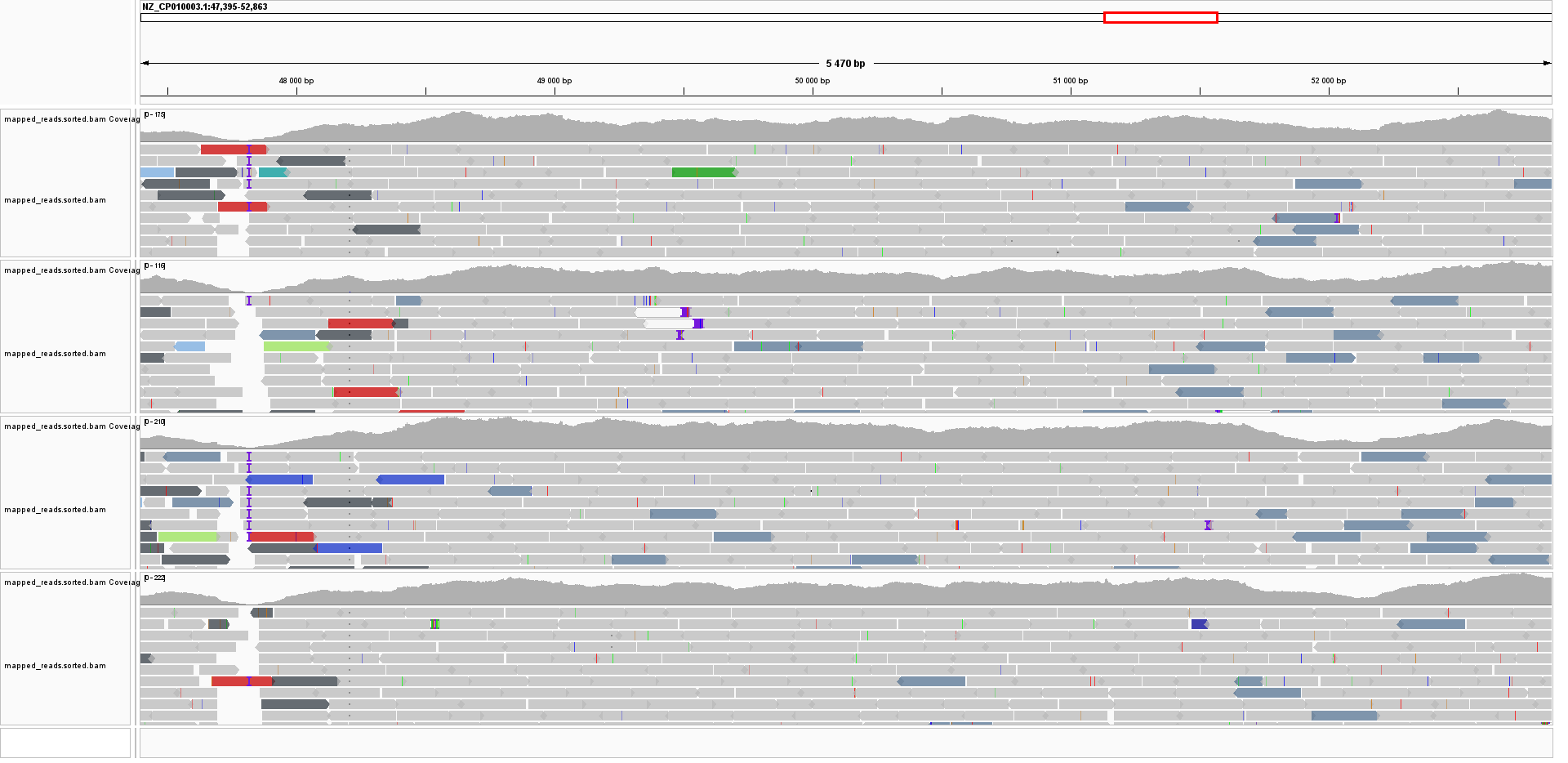


The observed window contains the *cry1Ab3* gene with 1000 bp surroundings on both sides. From top to bottom, the results of samples 1-4 are shown.

## Figure S28: Read mapping results for the metagenomic short reads at the location of the *cry1Ac5* gene.


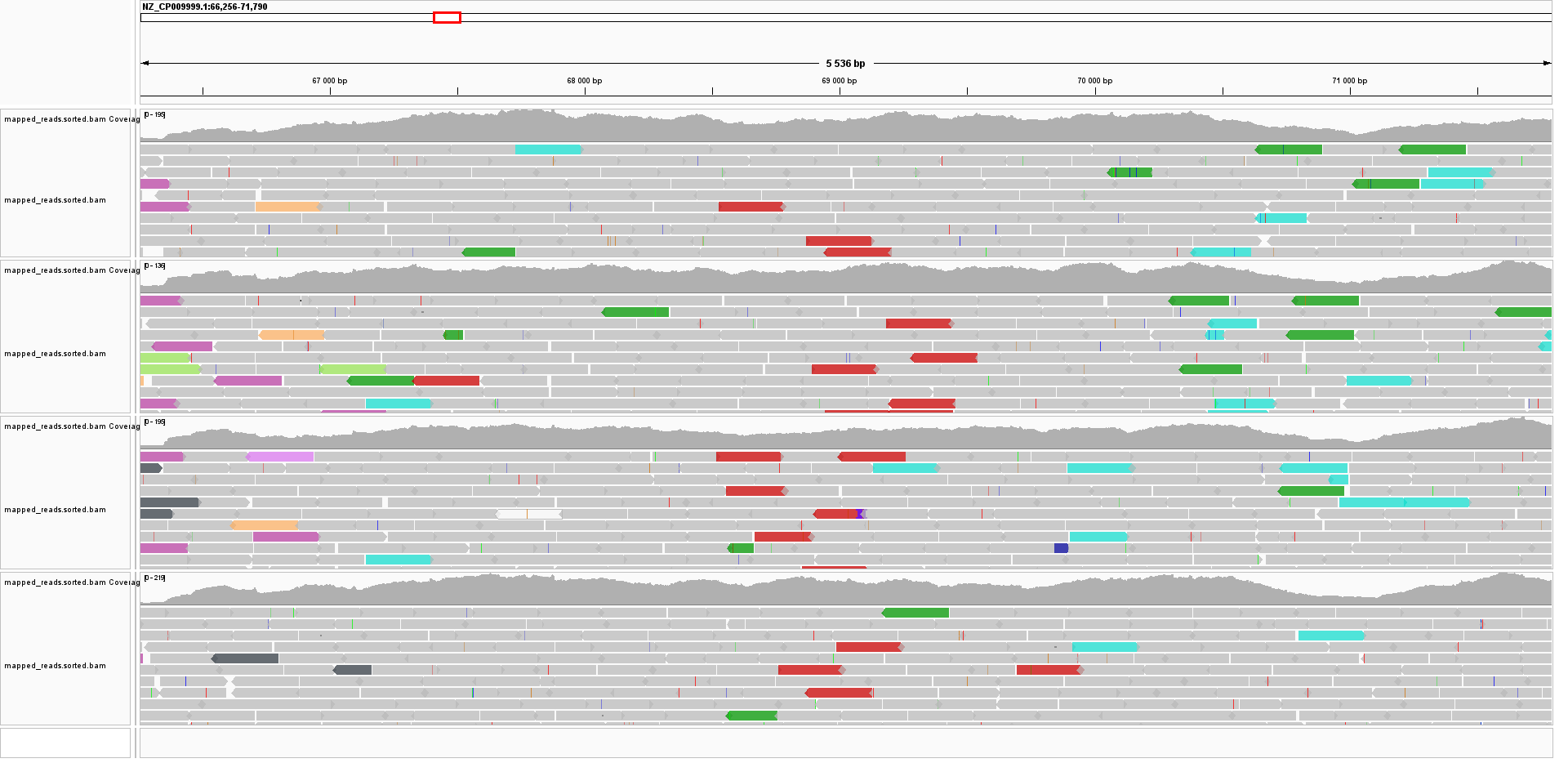


The observed window contains the *cry1Ac5* gene with 1000 bp surroundings on both sides. From top to bottom, the results of samples 1-4 are shown.

## Figure S29: Read mapping results for the metagenomic long reads at the location of the *cry1Aa8* gene.


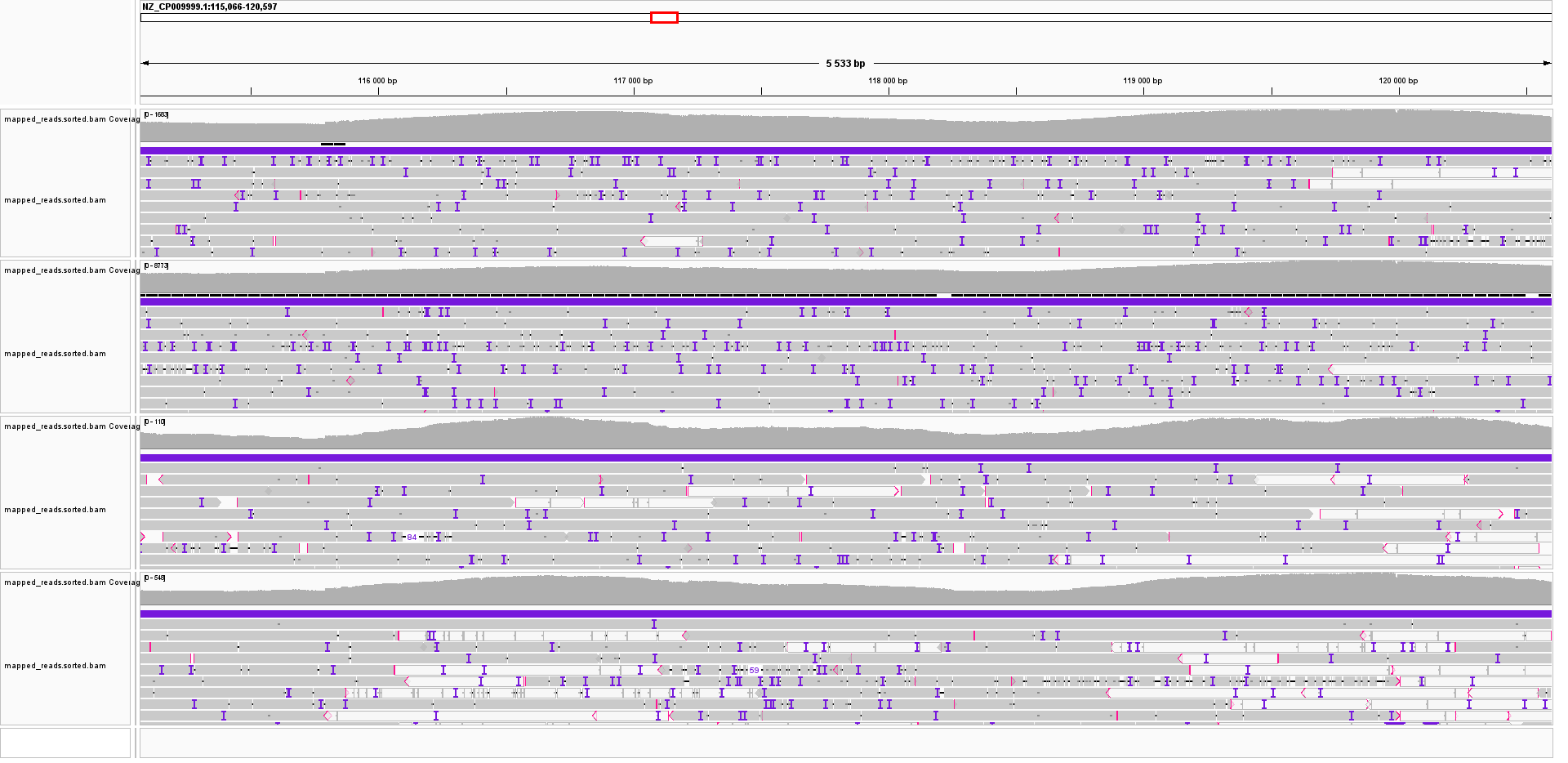


The observed window contains the *cry1Aa8* gene with 1000 bp surroundings on both sides. From top to bottom, the results of samples 1-4 are shown.

## Figure S30: Read mapping results for the metagenomic long reads at the location of the *cry1Ab3* gene.


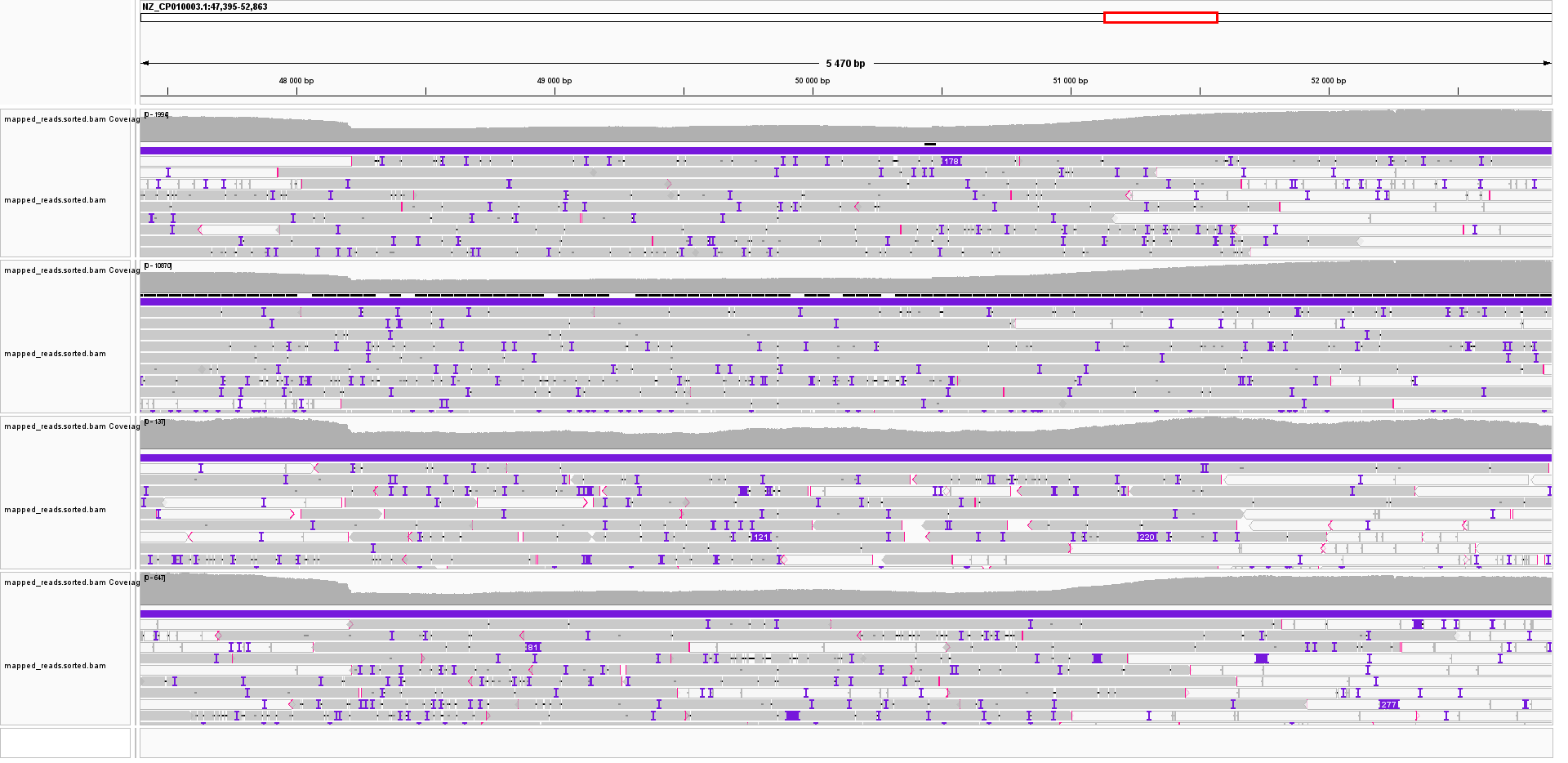


The observed window contains the *cry1Ab3* gene with 1000 bp surroundings on both sides. From top to bottom, the results of samples 1-4 are shown.

## Figure S31: Read mapping results for the metagenomic long reads at the location of the *cry1Ac5* gene.


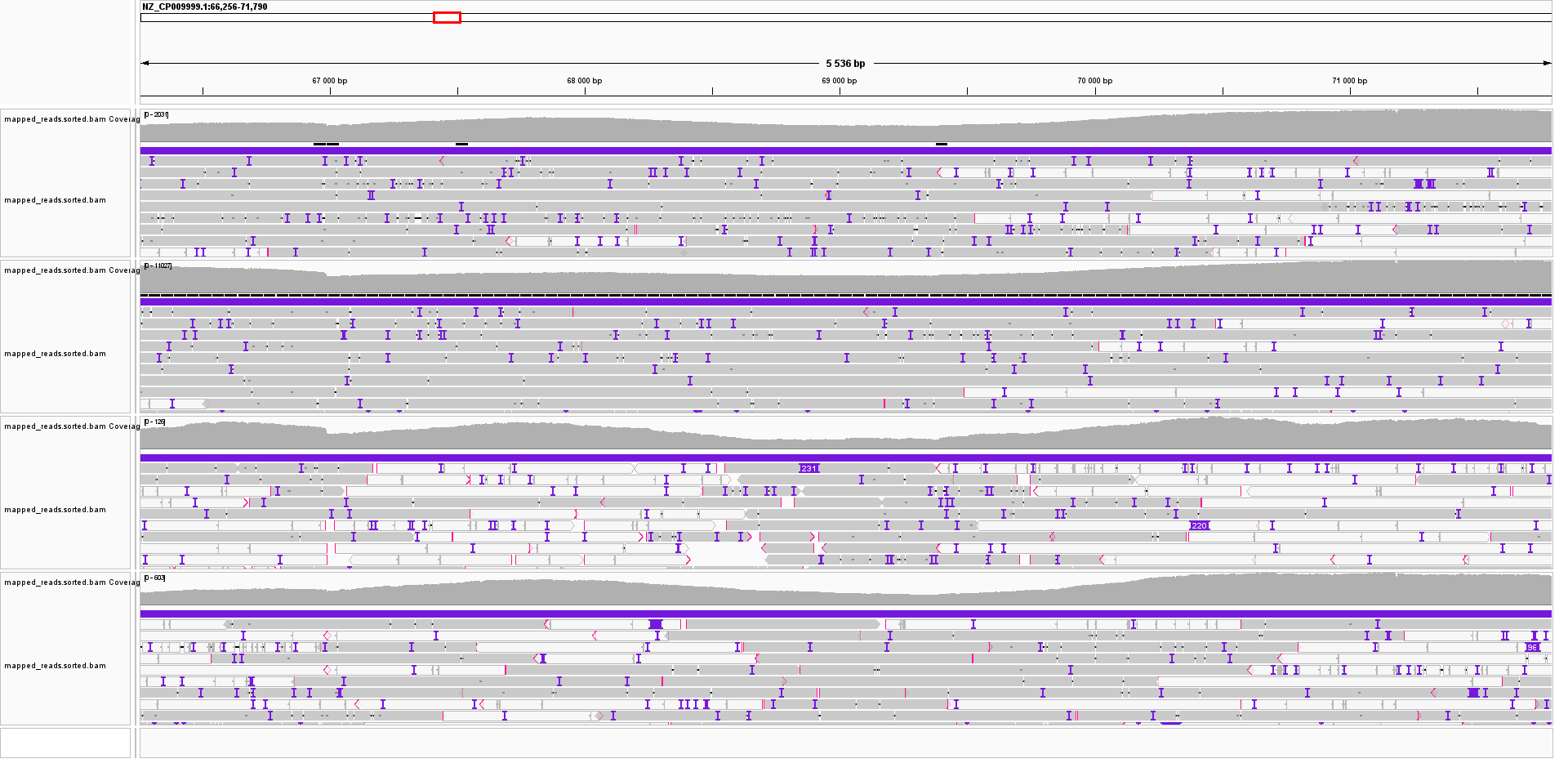


The observed window contains the *cry1Ac5* gene with 1000 bp surroundings on both sides. From top to bottom, the results of samples 1-4 are shown.

## Figure S32: Read mapping results for the metagenomic short and long reads at the location of the *cry1Aa8* gene in the hybrid assembly of sample 1.


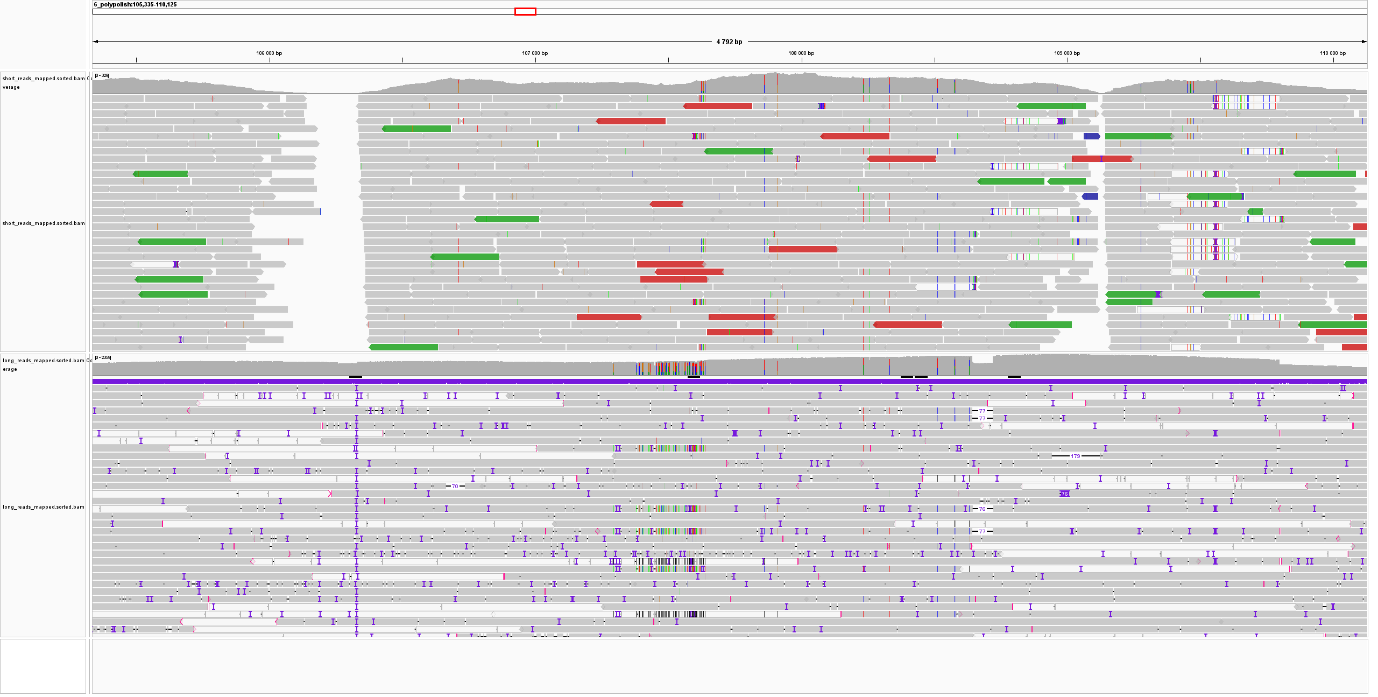


The observed window contains the *cry1Aa8* gene with 1000 bp surroundings on both sides. From top to bottom, the short and long reads are shown.

## Figure S33: Read mapping results for the metagenomic short and long reads at the location of the *cry1Ab3* gene in the hybrid assembly of sample 3.


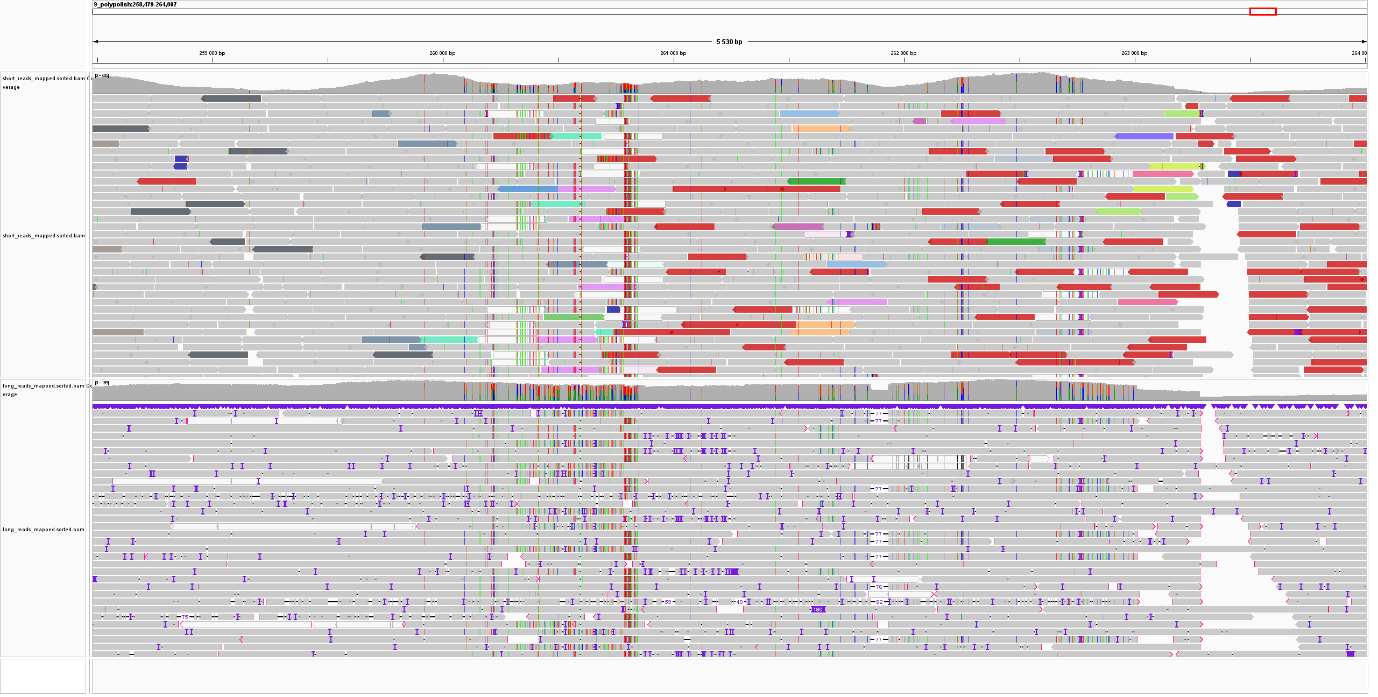


The observed window contains the *cry1Ab3* gene with 1000 bp surroundings on both sides. From top to bottom, the short and long reads are shown.

## Figure S34: Alignment of the Sanger sequence of isolate 2 of sample 4 against *cry1Ab3* and *cry1Ac5*


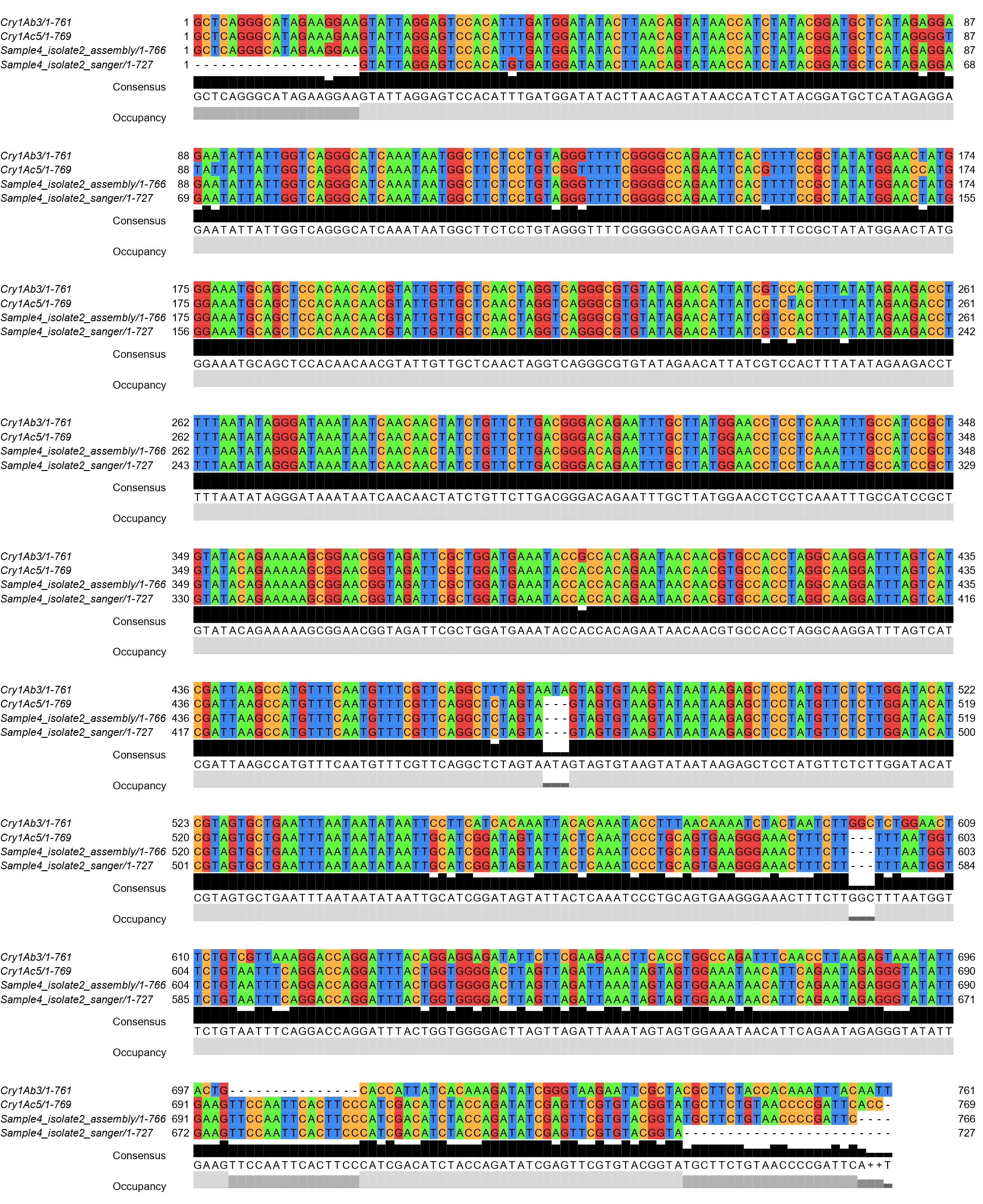


# Tables

## Table S1: Metazoan reference genomes included in the Kraken 2 database

| **Species** | **Accession** |
| --- | --- |
| *Aedes aegypti* | GCF_002204515 |
| *Aedes albopictus* | GCF_006496715 |
| *Anas platyrhynchos* | GCF_015476345 |
| *Stomoxys calcitrans* | GCF_001015335 |
| *Apis mellifera* | GCF_003254395 |
| *Bos taurus* | GCF_002263795 |
| *Capra hircus* | GCF_001704415 |
| *Cavia porcellus* | GCF_000151735 |
| *Chlorocebus sabaeus* | GCF_015252025 |
| *Columba livia* | GCF_000337935 |
| *Culex quinquefasciatus* | GCF_015732765 |
| *Equus caballus* | GCF_002863925 |
| *Gallus gallus* | GCF_000002315 |
| *Homo sapiens* | GCF_000001405 |
| *Ixodes scapularis* | GCF_002892825 |
| *Meleagris gallopavo* | GCF_000146605 |
| *Mesocricetus auratus* | GCF_000349665 |
| *Mus musculus* | GCF_000001635 |
| *Numida meleagris* | GCF_002078875 |
| *Ovis aries* | GCF_002742125 |
| *Rattus norvegicus* | GCF_015227675 |
| *Sus scrofa* | GCF_000003025 |

## Table S2: Read filtering statistics of the isolate sequencing data

| Sample | Isolate | Number of reads before filtering | Number of unorphaned reads after filtering |
| --- | --- | --- | --- |
| 1 | 1 | 1 586 180 | 1 467 176 |
|  | 2 | 2 002 340 | 1 844 286 |
|  | 3 | 1 968 574 | 1 809 692 |
|  | 4 | 2 153 838 | 1 948 160 |
| 2 | 1 | 1 602 450 | 1 449 294 |
|  | 2 | 1 780 488 | 1 651 988 |
|  | 3 | 1 444 822 | 1 299 336 |
|  | 4 | 1 818 218 | 1 666 622 |
| 3 | 1 | 2 935 538 | 2 761 032 |
|  | 2 | 2 261 328 | 2 057 678 |
|  | 3 | 1 712 102 | 1 527 154 |
|  | 4 | 1 977 770 | 1 813 876 |
| 4 | 1 | 1 913 294 | 1 735 242 |
|  | 2 | 1 922 308 | 1 751 194 |
|  | 3 | 1 850 596 | 1 707 546 |
|  | 4 | 2 059 918 | 1 884 612 |

## Table S3: Read filtering statistics of the metagenomic sequencing data

| **Sample** | Illumina | | | | ONT | | | | | |
| --- | --- | --- | --- | --- | --- | --- | --- | --- | --- | --- |
|  | Before filtering | | After filtering | | Before filtering | | | After filtering | | |
|  | Number of reads | Number of bases | Number of reads | Number of bases | Number of reads | Number of bases | Read length N50 | Number of reads | Number of bases | Read length N50 |
| 1 | 1 326 440 | 308 859 343 | 1 212 908 | 266 656 401 | 2 182 228 | 2 950 649 835 | 1 910 | 1 013 751 | 2 165 847 422 | 2 321 |
| 2 | 1 003 842 | 243 397 823 | 902 448 | 204 796 603 | 10 768 755 | 17 443 039 454 | 2 319 | 5 656 636 | 13 539 052 040 | 2 708 |
| 3 | 1 461 656 | 344 344 876 | 1 335 058 | 295 188 207 | 462 597 | 344 945 680 | 888 | 68 902 | 136 618 423 | 1 982 |
| 4 | 1 637 274 | 375 344 154 | 1 508 832 | 326 924 822 | 1 300 378 | 1 194 359 074 | 1 135 | 398 831 | 637 890 536 | 1 583 |

## Table S4: Assembly statistics for the isolate assemblies

| Sample | Isolate | Total length | Contig length N50 | Number of contigs |
| --- | --- | --- | --- | --- |
| 1 | 1 | 6 484 269 | 66 475 | 367 |
|  | 2 | 6 497 813 | 71 924 | 377 |
|  | 3 | 6 483 551 | 75 734 | 383 |
|  | 4 | 6 489 004 | 93 634 | 368 |
| 2 | 1 | 6 485 927 | 79 982 | 369 |
|  | 2 | 6 486 075 | 66 477 | 393 |
|  | 3 | 6 480 566 | 52 292 | 454 |
|  | 4 | 6 480 517 | 40 650 | 497 |
| 3 | 1 | 6 476 886 | 48 902 | 555 |
|  | 2 | 6 497 361 | 84 575 | 376 |
|  | 3 | 6 469 733 | 54 842 | 411 |
|  | 4 | 6 485 971 | 69 948 | 385 |
| 4 | 1 | 6 475 218 | 75 734 | 369 |
|  | 2 | 6 481 281 | 71 893 | 363 |
|  | 3 | 6 480 178 | 79 982 | 370 |
|  | 4 | 6 485 910 | 75 734 | 380 |

## Table S5: Assembly statistics for the metagenomic assemblies

| Sample | Assembler | Total length | Contig length N50 | Number of contigs |
| --- | --- | --- | --- | --- |
| 1 | Unicycler | 6 794 521 | 1 370 376 | 57 |
| 2 | Trycycler | 6 842 374 | 5 674 843 | 15 |
| 3 | Unicycler | 6 724 162 | 447 508 | 149 |
| 4 | Unicycler | 6 662 308 | 255 398 | 271 |

## Table S6: Amount of SNPs between the Trycycler assembly of sample 2 and the publicly available *B. thuringiensis* serovar *kurstaki* strain HD-1 assemblies determined by dnadiff

| **Accession** | **SNPs compared to Trycycler assembly** |
| --- | --- |
| GCA_000717535.1 | 4523 |
| GCA_000835235.1 | 44 |
| GCA_020809105.1 | 445 |
| GCA_000710255.1 | 39 |
| GCA_025210105.1 | 153 |

## Table S7: Amount of SNPs between all short read sequencing data and the publicly available *B. thuringiensis* serovar *kurstaki* strain HD-1 assemblies determined by the CFSAN SNP pipeline

| **Assembly** | **SNPs** |
| --- | --- |
| GCA_000717535.1 | 590 |
| GCA_000835235.1 | 10 |
| GCA_020809105.1 | 26 |
| GCA_000710255.1 | 11 |
| GCA_025210105.1 | 40 |

## Table S8: Completeness of the found AMR genes found in the isolate and metagenomic assemblies

| **Locus** | **Match Type** | **Description** |
| --- | --- | --- |
| *bla* | Mutant | 12 coding mutations |
| *satA* | Indel Truncation | 6 bp Insertion at 172, truncation at codon 186 (of 185 codons), 22 coding mutations |
| *blaIII* | Indel Nonstop | 9 bp Deletion at 28, 22 bp Insertion at 105, 1 bp Insertion at 125, 5 bp Deletion at 133, 1 bp Insertion at 528, 1 bp Deletion at 532, 43 coding mutations |
| *fosB/fosBx1* | Mutant | V60I/V60I |
| *bla2* | Mutant | 19 coding mutations |
| *vanZ-F* | Indel Truncation | 12 bp Insertion at 475, truncation at codon 207 (of 207 codons), 70 coding mutations |

## Table S9: Taxonomic classification of short and long reads of the metagenomic sequencing data

| Sample | Kraken2 classification (species-level) | |
| --- | --- | --- |
|  | Short reads (%) | Long reads (%) |
| 1 | *Bacillus thuringiensis* (52.78), *Bacillus cereus* (1.29) | *Bacillus thuringiensis* (80.65), *Bacillus cereus* (3.97),  *Bacillus mycoides* (1.02) |
| 2 | *Bacillus thuringiensis* (57.74), *Bacillus cereus* (1.57) | *Bacillus thuringiensis* (82.36), *Bacillus cereus* (3.90),  *Bacillus mycoides* (1.01) |
| 3 | *Bacillus thuringiensis* (53.92), *Bacillus cereus* (1.53) | *Bacillus thuringiensis* (79.63), *Bacillus cereus* (3.80),  *Bacillus mycoides* (1.02) |
| 4 | *Bacillus thuringiensis* (50.96), *Bacillus cereus* (1.36) | *Bacillus thuringiensis* (76.04), *Bacillus cereus* (4.59),  *Bacillus mycoides* (1.27) |

## Table S10: Structural variants with annotation in metagenomic sample 1

| **ref_chr** | **query_chr** | **size** | **SV_type** | **ref_anno** | **query_anno** |
| --- | --- | --- | --- | --- | --- |
| NZ_CP009999.1 | 6 | 172 | deletion_mobile | Pesticidal crystal protein Cry1Ag | Pesticidal crystal protein Cry1Ag |
| NZ_CP009999.1 | 6 | 200 | deletion_mobile | Pesticidal crystal protein Cry1Ag | Pesticidal crystal protein Cry1Ag |
| NZ_CP009999.1 | 6 | 231 | deletion_mobile | Pesticidal crystal protein Cry1Ag | / |
| NZ_CP010000.1 | 8 | 436 | deletion_mobile | NTTRR-F1 domain | Hypothetical protein |
| NZ_CP010001.1 | 9 | 166 | contraction | Cell surface protein | S-layer-like y domain-containing protein |
| NZ_CP010005.1 | 4 | 19782 | contraction | / | / |
| NZ_CP010005.1 | 4 | 151 | deletion_mobile | IS4 family transposase | IS4 family transposase |
| NZ_CP010005.1 | 4 | 247 | deletion_mobile | UV DNA damage repair endonuclease | UV DNA damage repair endonuclease |
| NZ_CP010005.1 | 4 | 224 | deletion_mobile | IS3 family ISBth10 transposase ORF A | / |
| NZ_CP010005.1 | 1 | 276 | deletion_qmobile | Methylthioribose-1-phosphate isomerase 2 | Methylthioribose-1-phosphate isomerase 2 |
|  |  |  |  | IS4 family IS231C transposase | IS4 family transposase |
| NZ_CP010005.1 | 1 | 204 | deletion_mobile | IS4 family IS231C transposase | / |
| NZ_CP010005.1 | 1 | 68 | deletion_mobile | Glycine-rich cell wall structural protein 1-8-like | Hydrolase |
| NZ_CP010005.1 | 2 | 335 | deletion_mobile | IS110 family ISBth13 transposase | IS110 family ISBth13 transposase |
| NZ_CP010005.1 | 2 | 306 | deletion_mobile | 16S ribosomal RNA | 16S ribosomal RNA |
| NZ_CP010005.1 | 2 | 171 | deletion_mobile | 16S ribosomal RNA | / |

The first column gives the replicon of the reference genome where the structural variant was found, the second column gives the contig of the hybrid assembly. The third and fourth columns respectively show the size and type of the structural variant according to MUM&Co. The fifth and sixth columns show the annotations of the reference genome and the hybrid assembly respectively. The first three structural variants are located in and around the *cry1Aa8* gene. As mentioned in Section 3.3.5, there is likely a mis-assembly in the region of the *cry1Aa8* gene in the Unicycler assembly of sample 1.

## Table S11: Structural variants with annotation in metagenomic sample 3

| **ref_chr** | **query_chr** | **size** | **SV_type** | **ref_anno** | **query_anno** |
| --- | --- | --- | --- | --- | --- |
| NZ_CP010001.1 | 14 | 54 | deletion_mobile | NTTRR-F1 domain | Hypothetical protein |
| NZ_CP010001.1 | 14 | 166 | contraction | Cell surface protein | S-layer-like y domain-containing protein |
| NZ_CP010005.1 | 5 | 272723 | contraction | / | / |
| NZ_CP010005.1 | 3 | 68 | deletion_mobile | Glycine-rich cell wall structural protein 1-8-like | Hypothetical protein |
| NZ_CP010005.1 | 13 | 203 | contraction | (pseudo) putative membrane protein YhgE, phage infection protein (PIP) family | (pseudo) putative membrane protein YhgE, phage infection protein (PIP) family |
| NZ_CP010005.1 | 4 | 57 | deletion_mobile | Cell wall anchor protein | Hypothetical protein |

The first column gives the replicon of the reference genome where the structural variant was found, the second column gives the contig of the hybrid assembly. The third and fourth columns respectively show the size and type of the structural variant according to MUM&Co. The fifth and sixth columns show the annotations of the reference genome and the hybrid assembly respectively.

## Table S12: Structural variants with annotation in metagenomic sample 4

| **ref_chr** | **query_chr** | **size** | **SV_type** | **ref_anno** | **query_anno** |
| --- | --- | --- | --- | --- | --- |
| NZ_CP010005.1 | 4 | 306 | deletion_mobile | Hypothetical protein | Hypothetical protein |
| NZ_CP010005.1 | 2 | 68 | deletion_mobile | Glycine-rich cell wall structural protein 1-8-like | Hypothetical protein |
| NZ_CP010005.1 | 14 | 76 | deletion_novel | NprRB | Hypothetical protein |
| NZ_CP010005.1 | 47 | 10420 | contraction | / | / |

The first column gives the replicon of the reference genome where the structural variant was found, the second column gives the contig of the hybrid assembly. The third and fourth columns respectively show the size and type of the structural variant according to MUM&Co. The fifth and sixth columns show the annotations of the reference genome and the hybrid assembly respectively.

## Table S13: Insecticidal gene content of the publicly available *B. thuringiensis* serovar *kurstaki* strain HD-1 genomes

| name | *cry1Aa* | *cry1Ab* | *cry1Ac* | *cry1Ia* | *cry2Aa* | *cry2Ab* | *vip3Aa* |
| --- | --- | --- | --- | --- | --- | --- | --- |
| Sample 2 | 0 | 0 | 0 | 0 | 0 | 0 | 0 |
| GCA_000710255 | Missing | 0 | Contig edge | 0 | 0 | 0 | 0 |
| GCA_000717535 | 15 | 0 | 0 | 0 | 12 | 3 | 0 |
| GCA_000835235 | 0 | 0 | 0 | 0 | 0 | 0 | 0 |
| GCA_020809105 | 0 | 3 | 0 | 0 | 0 | 0 | 0 |
| GCA_025210105 | Contig edge | Contig edge | Contig edge | 0 | 0 | 0 | 0 |

This table shows the number of SNPs between each of the publicly available *B. thuringiensis* serovar *kurstaki* strain HD-1 genomes and the insecticidal genes in the Trycycler assembly of sample 2.

# Text

## S1: Detailed description of the long-read first hybrid assembly approach of sample 2

Stricter filtering was employed for the hybrid assembly of sample 2. Long reads were filtered with NanoFilt 2.8.0 to remove reads shorter than 6000 bp and with a mean Phred score of less than 15. The filtered reads were then subsampled in 24 sets using the Trycycler subsample function with an estimated genome size of 6.8 Mb. The first 8 read subsets were assembled using miniasm 0.3r179 (Li, 2016), using the miniasm_and_minipolish.sh script available in Minipolish. Minipolish 0.1.3 (Wick and Holt, 2019), minimap2 2.24, and Racon 1.3.1 were used as dependencies. The next 8 read subsets were assembled using Canu 2.1.1 (Koren et al., 2017) with the useGrid parameter set to false and the genomeSize parameter set to 6.8 Mb. The final 8 read subsets were assembled using Flye 2.9.1 (Kolmogorov et al., 2019) using the --nano-hq option and with the --genome-size parameter set to 6.8 Mb. Clustering of the obtained contigs was done as described in the Trycycler manual, resulting in 48 clusters. Clusters with only one contig were immediately removed. The remaining clusters were attempted to be reconciled as circular plasmids using the Trycycler reconcile function. For clusters where this failed, the contig that prevented circular reconciliation was removed until the cluster was empty or successfully reconciled. If reconciling as a circular plasmid failed, reconciliation as a linear plasmid was attempted using the same procedure where contigs that prevented reconciliation were removed. After the reconciliation process, 30 clusters remained, 13 of which were circular. Using strict filtering when using Trycycler can impair the recovery of small plasmids (Wick et al., 2023). To ensure no small plasmids were missed, an additional hybrid assembly was made with Unicycler 0.5.0 with the same dependencies listed in Section 2.5.2. This was done using the less strictly filtered reads mentioned above (only reads shorter than 1000bp and with a mean Phred score of less than 7 were removed) to enable the recovery of small plasmids. This resulted in 16 contigs, 12 of which were circular. To determine which of the Unicycler contigs are redundant, they were mapped against a collection of Trycycler contigs containing one contig per cluster using minimap2 with the asm5 preset. Only one of the Unicycler contigs didn’t map to the collection of Trycycler contigs. This Unicycler contig was added as a new cluster to Trycycler. To determine which of the linear Trycycler clusters were just parts of larger circular plasmids, a collection of linear Trycycler contigs containing one contig per linear cluster, was mapped against a collection of circular Trycycler contigs, containing one contig per circular cluster using minimap2 with the asm5 preset. Two of the 17 linear contigs did not map to any circular contigs. These two linear contigs successfully mapped to each other, so the cluster represented by the largest of these two was kept. All other linear clusters were discarded, leaving 13 circular Trycycler clusters, 1 circular Unicycler cluster, and 1 linear Trycycler cluster. Multiple sequence alignment was performed on each of these clusters using the Trycycler msa function. Next, reads were partitioned, i.e. reads were assigned to the cluster they best aligned to. For the read partitioning, the less strictly filtered long reads were used (only reads shorter than 1000bp and with a mean Phred score of less than 7 were removed) as recommended in the Trycycler manual. After read partitioning, a consensus sequence was generated using the Trycycler consensus function. Assembly polishing was done in the same way as for the other metagenomic assemblies. For short read polishing, the short reads that mapped to the Trycycler consensus sequence during the read partitioning step were used.

## S2: Literature overview of *B. thuringiensis* serovar *kurstaki* strain HD-1 genomes and their insecticidal gene content

A detailed characterization requires a high-quality, closely related reference genome. A reference genome that is too distant will cause more false positives, ambiguously called sites, and gaps, providing less reliable results (Pightling et al., 2014). To find the most suitable reference genome, five publicly available assemblies were compared to our sequencing data as described in Section 2.5.3. The insecticidal gene content of all five assemblies was evaluated. The results can be seen in Supplementary Table S13.

As can be seen in Supplementary Tables S6 and S7, our sequencing data was most closely related to GCF_000710255 and GCF_000835235. GCF_000710255 serves as the primary U.S. reference standard for all commercial insecticidal formulations of B. thuringiensis manufactured around the world (Day et al., 2014). This genome assembly is quite fragmented however, so we opted to choose GCF_000835235 as a reference genome since it is a more complete assembly. GCF_000710255 and GCF_000835235 both contain *cry1Ab*, *cry1Ia*, *cry2Aa*, *cry2Ab*, and *vip3Aa* genes that are entirely identical, down to the nucleotide level. GCF_000710255 misses the *cry1Aa* gene. This gene is present in all the other assemblies, so it’s probably missing because of the fragmented nature of the assembly. It also only contains a partial *cry1Ac* gene on a contig edge with no mutations in the part that is present.

According to the EFSA risk assessment, *B. thuringiensis* serovar *kurstaki* strain HD-1 contains genes encoding five insecticidal crystal proteins: Cry1Aa, Cry1Ab, Cry1Ac, Cry2Aa, and Cry2Ab. In the literature, the gene encoding the Cry1Aa1 protein can be found in this strain (Schnepf et al., 1985). However, the Cry1Aa-encoding gene in our samples has 2 SNPs of difference with the Cry1Aa1-encoding gene. We find the gene encoding the Cry1Aa8 protein instead. This gene is associated with *B. thuringiensis strain EMCC-0073* instead of *B. thuringiensis* serovar *kurstaki* strain HD-1 (CHI-LI, 1996). Our samples also contain the gene encoding the Cry1Ab3 protein. This gene was previously found in *B. thuringiensis* serovar *kurstaki* strain HD-1 (Geiser et al., 1986). The gene encoding the Cry1Ab4 protein was also found in *B. thuringiensis* serovar *kurstaki* strain HD-1 (Kondo et al., 1987). This protein has the exact same amino acid sequence compared to Cry1Ab3, but the genes differ in one nucleotide in the coding region. Our sequencing data also contains the gene encoding the Cry1Ac5 protein. This gene is associated with *B. thuringiensis* serovar *kurstaki* strain PS81GG (GenBank accession AAA22339.1). In literature, the gene encoding the Cry1Ac13 protein has been found in *B. thuringiensis* serovar *kurstaki* strain HD-1 (Qiao et al., 1993). This is however only a partial protein sequence. This part is also present in Cry1Ac5. According to the EFSA risk assessment, *B. thuringiensis* serovar *kurstaki* strain HD-1 does not contain a gene for a Cry1Ia protein, but the gene for Cry1Ia3 has been found in *B. thuringiensis* serovar *kurstaki* strain HD-1 (Shin et al., 1995). We found a different gene in our samples, the one for Cry1Ia10. We also found the gene encoding the Cry2Aa1 protein, which has been found before in *B. thuringiensis* serovar *kurstaki* strain HD-1 (Donovan et al., 1988). The gene encoding Cry2Aa2 has the same coding sequence. We also found the gene encoding the Cry2Ab1 protein in our data, this gene has been found before in *B. thuringiensis* serovar *kurstaki* strain HD-1 (Widner and Whiteley, 1989). This gene has an identical coding region compared to the Cry2Ab2 encoding gene. Finally, we also found a gene encoding the Vip3Aa58 protein. This gene has also previously been found in *B. thuringiensis* serovar *kurstaki* strain HD-1 (Baranek et al., 2015).

## S3: Evaluation of sequencing quality

Raw and filtered read counts for the isolate WGS data are listed in Supplementary Table S2. After filtering, the median read count for all isolates was 1 743 218. Read statistics for the metagenomic sequencing data are listed in Supplementary Table S3. After filtering, the median read count for the short reads was 1 273 983. For the filtered long reads, the median read count was 706 291, the median base count was 1 401 868 979 and the median N50 was 2 151.5. An overview of the assembly statistics for the isolate assemblies is provided in Supplementary Table S4 and an overview of the assembly statistics for the metagenomic assemblies is provided in Supplementary Table S5.

## S4: PCR amplification and Sanger sequencing of the *cry1Ac5* gene in isolate 2 in sample 4

The insecticidal gene detection and the SNP pipeline both indicated that there were SNPs in the *cry1Ac5* gene of isolate 2 of sample 4. An additional PCR amplification followed by Sanger sequencing was performed to find out whether or not this was a sequencing artefact. A multiple sequence alignment was done, comparing a part of the genome assembly of isolate 2 of sample 4, the sanger sequence and the nucleotide sequences encoding Cry1Ab3 and CryAc5. The result of this can be seen in Supplementary Figure S34. The alignment shows that the genome assembly fragment and the Sanger sequence are identical, except for one SNP on position 37 of the genome assembly fragment. This is likely a sequencing error in the Sanger sequence. The alignment also shows that both sequences of isolate 2 of sample 4 correspond to the *cry1Ab3* gene in the first half. At around 300 basepairs, there is a region that is identical in both insecticidal genes. After this region, the sequences of isolate 2 of sample 4 correspond to the *cry1Ac5* gene.

# References

Baranek, J., Kaznowski, A., Konecka, E., and Naimov, S. (2015). Activity of vegetative insecticidal proteins Vip3Aa58 and Vip3Aa59 of Bacillus thuringiensis against lepidopteran pests. *J Invertebr Pathol* 130, 72–81. doi: 10.1016/j.jip.2015.06.006

CHI-LI, L. I. U. (1996). Bacillus thuringiensis isolates active against lepidopteran pests. Available at: https://lens.org/079-074-250-599-844

Day, M., Ibrahim, M., Dyer, D., and Bulla, L. (2014). Genome Sequence of Bacillus thuringiensis subsp. *kurstaki* Strain HD-1. *Genome Announc* 2. doi: 10.1128/genomeA.00613-14

Donovan, W. P., Dankocsik, C. C., Gilbert, M. P., Gawron-Burke, M. C., Groat, R. G., and Carlton, B. C. (1988). Amino acid sequence and entomocidal activity of the P2 crystal protein. An insect toxin from Bacillus thuringiensis var. kurstaki. *Journal of Biological Chemistry* 263, 561–567. doi: 10.1016/S0021-9258(19)57428-2

Geiser, M., Schweitzer, S., and Grimm, C. (1986). The hypervariable region in the genes coding for entomopathogenic crystal proteins of Bacillus thuringiensis: nucleotide sequence of the kurhd1 gene of subsp. kurstaki HD1. *Gene* 48, 109–118. doi: 10.1016/0378-1119(86)90357-4

Kolmogorov, M., Yuan, J., Lin, Y., and Pevzner, P. A. (2019). Assembly of long, error-prone reads using repeat graphs. *Nat Biotechnol* 37, 540–546. doi: 10.1038/s41587-019-0072-8

Kondo, S. I., Tamura, N., Kunitate, A., Hattori, M., Akashi, A., and Ohmori, I. (1987). Cloning and nucleotide sequencing of two insecticidal δ- endotoxin genes from bacillus thuringiensis var. Kurstaki hd-1 dna. *Agric Biol Chem* 51, 455–463. doi: 10.1080/00021369.1987.10868034

Koren, S., Walenz, B. P., Berlin, K., Miller, J. R., Bergman, N. H., and Phillippy, A. M. (2017). Canu: scalable and accurate long-read assembly via adaptive *k* -mer weighting and repeat separation. *Genome Res* 27, 722–736. doi: 10.1101/gr.215087.116

Li, H. (2016). Minimap and miniasm: fast mapping and de novo assembly for noisy long sequences. *Bioinformatics* 32, 2103–2110. doi: 10.1093/bioinformatics/btw152

Pightling, A. W., Petronella, N., and Pagotto, F. (2014). Choice of Reference Sequence and Assembler for Alignment of Listeria monocytogenes Short-Read Sequence Data Greatly Influences Rates of Error in SNP Analyses. *PLoS One* 9, e104579. doi: 10.1371/journal.pone.0104579

Qiao, L., Tian, Y., and Mang, K. (1993). [Nucleotide sequence of the toxic domain of an insecticidal protein gene from B. thuringiensis subsp. kurstaki HD-1]. *Wei Sheng Wu Xue Bao* 33, 383–6.

Schnepf, H. E., Wong, H. C., and Whiteley, H. R. (1985). The amino acid sequence of a crystal protein from Bacillus thuringiensis deduced from the DNA base sequence. *Journal of Biological Chemistry* 260, 6264–6272. doi: 10.1016/S0021-9258(18)88966-9

Shin, B. S., Park, S. H., Choi, S. K., Koo, B. T., Lee, S. T., and Kim, J. I. (1995). Distribution of cryV-type insecticidal protein genes in Bacillus thuringiensis and cloning of cryV-type genes from Bacillus thuringiensis subsp. kurstaki and Bacillus thuringiensis subsp. entomocidus. *Appl Environ Microbiol* 61, 2402–2407. doi: 10.1128/aem.61.6.2402-2407.1995

Wick, R. R., and Holt, K. E. (2019). Benchmarking of long-read assemblers for prokaryote whole genome sequencing. *F1000Res* 8, 2138. doi: 10.12688/f1000research.21782.1

Wick, R. R., Judd, L. M., and Holt, K. E. (2023). Assembling the perfect bacterial genome using Oxford Nanopore and Illumina sequencing. *PLoS Comput Biol* 19, e1010905. doi: 10.1371/journal.pcbi.1010905

Widner, W. R., and Whiteley, H. R. (1989). Two highly related insecticidal crystal proteins of Bacillus thuringiensis subsp. kurstaki possess different host range specificities. *J Bacteriol* 171, 965–974. doi: 10.1128/jb.171.2.965-974.1989
